# Positionspapier der ÖGR und ÖGP zur Diagnose und Therapie der Sarkoidose 2024

**DOI:** 10.1007/s00508-024-02444-z

**Published:** 2024-10-09

**Authors:** Georg Sterniste, Klaus Hackner, Florentine Moazedi-Fürst, Marie Grasl, Marco Idzko, Guangyu Shao, Claudia Guttmann-Ducke, Emina Talakić, Helmut Prosch, Sylvia Lohfink-Schumm, Michael Gabriel, Clarice Lim, Johann Hochreiter, Brigitte Bucher, Barbara C Böckle, Hans Peter Kiener, Christina Duftner, Kastriot Kastrati, Eva Rath, Marion Funk, Judith Löffler-Ragg, Monika Steinmaurer, Gabor Kovacs, Nicolas Verheyen, Holger Flick, Marlies Antlanger, Gerhard Traxler, Elisabeth Tatscher, Ralf Harun Zwick, David Lang

**Affiliations:** 1 Abteilung für Innere Medizin und Pneumologie, Klinik Floridsdorf, 1210 Wien, Österreich; 2 Klinische Abteilung für Pneumologie, Universitätsklinikum Krems, Karl Landsteiner Privatuniversität für Gesundheitswissenschaften, 3500 Krems, Österreich; 3 Klinische Abteilung für Rheumatologie und Immunologie, Medizinische Universität Graz, 8036 Graz, Österreich; 4 Abteilung für Atemwegs- und Lungenkrankheiten, Klinik Penzing, Ludwig Boltzmann Institut für Lungengesundheit, Wien, Österreich, 1140 Wien, Österreich; 5 Univ. Klinik für Innere Medizin II, Klin. Abteilung für Pulmologie, Medizinische Universität Wien, Wien, Österreich; 6 Klinische Abteilung für Allgemeine Radiologische Diagnostik, Universitätsklinik für Radiologie, Medizinische Universität Graz, Graz, Österreich; 7 Universitätsklinikum für Innere Medizin 4/Pneumologie, Kepler Universitätsklinikum, Johannes Kepler Universität, Linz, Österreich; 8 Univ. Klinik für Radiologie und Nuklearmedizin, Medizinische Universität Wien, Wien, Österreich; 9 Institut für Pathologie und Molekularpathologie, Kepler Universitätsklinikum, Johannes Kepler Universität, Linz, Österreich; 10 Institut für Nuklearmedizin und Endokrinologie, Kepler Universitätsklinikum, Johannes Kepler Universität, Linz, Österreich; 11 Lungenfibrose Forum Austria, Innermanzing, Österreich; 12 Abteilung Pneumologie, LKH Hochzirl Natters, Natters, Medizinische Universität Innsbruck, Innsbruck, Österreich; 13 Universitätsklinik für Dermatologie, Venerologie & Allergologie, Medizinische Universität Innsbruck, Innsbruck, Österreich; 14 Universitätsklinik für Innere Medizin III, Klinische Abteilung für Rheumatologie, Medizinische Universität Wien, Wien, Österreich; 15 Universitätsklinik für Innere Medizin II, Medizinische Universität Innsbruck, Innsbruck, Österreich; 16 1. Medizinische Abteilung, Hanusch Krankenhaus, Heinrich-Collin-Str. 30, 1140 Wien, Österreich; 17 Universitätsklinik für Augenheilkunde und Optometrie, Medizinische Universität Wien, Wien, Österreich; 18 Abteilung für Lungenkrankheiten, Klinikum Wels-Grieskirchen, 4600 Wels, Österreich; 19 Universitätsklinik für Innere Medizin, Klinische Abteilung für Pulmonologie, Medizinische Universität Graz, Graz, Österreich; 20 Universitätsklinik für Innere Medizin, Klinische Abteilung für Kardiologie, Medizinische Universität Graz, Graz, Österreich; 21 Universitätsklinik für Innere Medizin 2, Kepler Universitätsklinikum, Johannes Kepler Universität, Linz, Österreich; 22 Universitätsklinik für Neurologie, Kepler Universitätsklinikum, Johannes Kepler Universität, Linz, Österreich; 23 Universitätsklinik für Innere Medizin, Klinische Abteilung für Gastroenterologie und Hepatologie, Medizinische Universität Graz, Graz, Österreich; 24 Ambulante Rehabilitation, Ludwig Boltzmann Institute for Rehabilitation Research, Therme Wien Med, Wien, Österreich

**Keywords:** Glucocorticosteroide, Infliximab, Kardiale Magnetresonanztomographie, PET/CT, Niere, Glucocorticosteroids, Infliximab, Cardiac magnetic resonance imaging, PET/CT, Kidney

## Abstract

Die Sarkoidose ist in vielen Fällen eine Multisystemerkrankung, die eine interdisziplinäre medizinische Zusammenarbeit in Diagnostik, Therapie und in der medizinischen Betreuung im Verlauf erfordert. Aufgrund des oft chronischen Verlaufes ist es besonders wichtig, Patientinnen und Patienten mit ihren Prioritäten und Wünschen frühzeitig und umfassend in die medizinische Betreuung einzubinden und, wenn möglich, ein „shared decision making“ zu etablieren. Beim Verfassen dieses gemeinsamen Positionspapieres war es der Expertengruppe für interstitielle Lungenerkrankungen und „orphan diseases“ der Österreichischen Gesellschaft für Pneumologie sowie der Arbeitsgruppe Rheuma und Lunge der Österreichischen Gesellschaft für Rheumatologie und Rehabilitation ein besonderes Anliegen, sowohl PatientInnenvertreter als auch ExpertInnen für seltenere Organmanifestationen der Sarkoidose einzubeziehen. Dieses Positionspapier soll nicht nur ein Spiegel der aktuellen klinischen und wissenschaftlichen Praxis sein, sondern auch die nationale Expertise bündeln und durch Vernetzung und Austausch ein erster Schritt zu einer Stärkung der Betreuungsstruktur von PatientInnen mit Sarkoidose sein.

## Abstract (en)

In many cases sarcoidosis is a multisystemic disease that requires interdisciplinary medical cooperation in the diagnostics, treatment and medical care during follow-up. Due to the often chronic course, it is of utmost importance to include patients with their priorities and wishes at an early stage and extensively in disease management and to establish a shared decision making whenever possible. In the process of writing this joint position paper, the expert group on interstitial and orphan lung diseases of the Austrian Society for Pulmonology and the working group on rheumatological lung disorders of the Austrian Society for Rheumatology and Rehabilitation sought to include patient advocacy groups as well as experts for rare organ manifestations of sarcoidosis. This position paper is not only meant to reflect current scientific and clinical standards but should also focus the national expertise and by networking and exchange to be a first step to strengthen cooperation between stakeholders to ultimately improve care for patients with sarcoidosis.

## Einleitung, Definition, Epidemiologie

Dieses Positionspapier der Österreichischen Gesellschaft für Pneumologie (ÖGP) und Österreichischen Gesellschaft für Rheumatologie (ÖGR) richtet sich an alle medizinischen Fachdisziplinen. Interdisziplinäres Ziel ist es, einen aktuellen Überblick über die Epidemiologie, Pathogenese, Klinik, Diagnostik, Organmanifestationen und Therapiemöglichkeiten der Sarkoidose zu geben.

Die Sarkoidose ist eine komplexe, polyätiologische, inflammatorisch-granulomatöse Multisystemerkrankung, deren Genese noch nicht vollständig geklärt ist. In bis zu 95 % aller Fälle liegt eine pulmonale Beteiligung vor. Das klinische Bild ist sehr variabel und reicht von asymptomatischen Verläufen bis hin zur manifesten Organschädigung [1–3].

Die Prävalenzraten variieren in Abhängigkeit von Alter, Geschlecht, Ethnizität und Geografie: Weltweite Angaben reichen von 2,2 pro 100.000 Einwohner in Taiwan bis zu 46 pro 100.000 in Deutschland [4–6]. Daten zur Prävalenz in Österreich liegen bis dato nicht vor. Das durchschnittliche Erkrankungsalter liegt bei 46 ± 15 Jahren [7], Frauen sind tendenziell häufiger und in höherem Lebensalter von Sarkoidose betroffen als Männer [2].

## Diagnostik der Sarkoidose

### Histopathologische Untersuchung

In vielen Fällen wird eine Biopsie angestrebt, auch um andere Differenzialdiagnosen wie maligne Erkrankungen und Infektionen auszuschließen. Die Probe sollte von einer möglichst gut zugänglichen, betroffenen Stelle entnommen werden (z. B. Haut oder Lymphknoten) [8].

**Abb. 1:**
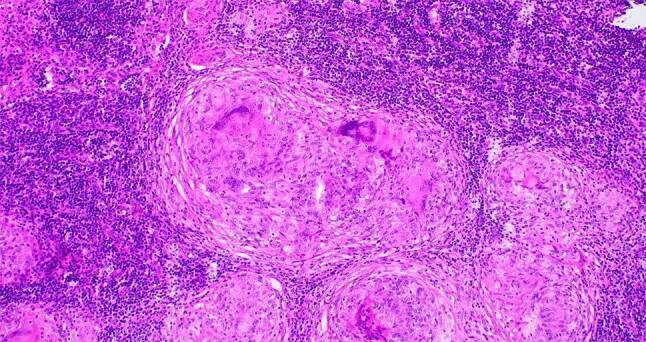
Morphologie des Sarkoidosegranuloms: kompakte, gut geformte Ansammlungen von großen, epitheloiden Makrophagen und vielkernigen Riesenzellen

Das histologische Markenzeichen der Sarkoidose ist das Granulom: Es ist meistens nicht nekrotisch, hat einen zentralen Kern aus Makrophagenaggregaten und vielkernigen Riesenzellen und eine äußere Schicht aus locker organisierten Lymphozyten, dendritischen Zellen und gelegentlich B‑Zellen [9]. Bei Unklarheiten sollte eine Färbung auf säurefeste Stäbchen, eine Kultur auf Pilze und Mykobakterien und eine *Mycobacterium-tuberculosis*-PCR erfolgen (Abb. 1).

### Bronchoalveoläre Lavage

Die bronchoalveoläre Lavage (BAL) ist ein relativ einfaches, risikoarmes bronchoskopisches Verfahren, wobei eine Lymphozytose > 15 % und ein CD4:CD8-Verhältnis > 3,5 die Diagnose einer pulmonalen Sarkoidose unterstützt [10, 11]. Daneben sollte am gewonnenen Material auch eine Färbung auf säurefeste Stäbchen, eine konventionelle Kultur auf Bakterien und Pilze, eine Mykobakterienkultur und eine *Mycobacterium-tuberculosis*-PCR durchgeführt werden, um eine Infektion als alternative Diagnose auszuschließen.

### Blutbasierte Diagnostik

Es existiert kein sicherer diagnostischer Laborbiomarker zur Erkennung der Sarkoidose oder ihrer Organmanifestationen, Laborbefunde können aber die Abklärung und Verlaufskontrollen gut komplementieren. Die Tab. 1 gibt aktuelle Empfehlungen hierzu wieder [4, 8, 12, 13]. Die höchste Evidenz besteht für die Testung von Serumkalzium initial und auch im Verlauf, da eine Hyperkalzämie unmittelbare therapeutische Konsequenzen nach sich zieht [8, 14].

**Tab. 1:** Empfehlungen zur Labordiagnostik der Diagnosestellung der Sarkoidose und bei Verlaufskontrollen. (Nach Crouser ED et al. [8])

Baseline-Screening		Verlaufskontrollen bei unauffälligem Baseline-Screening	
**Untersuchung**	**Indikation**	**Untersuchung**	**Intervall/Indikation**
Serumkreatinin, Serumkalzium	Nierenbeteiligung/Hyperkalzämie	Serumkreatinin, Serumkalzium	Jährlich
Leberfunktionsparameter	Leberbeteiligung	Leberfunktionsparameter	Jährlich
Blutbild	Differenzialdiagnostik	–	–
*Optional:* ACE, löslicher IL-2-Rezeptor, Neopterin	Differenzialdiagnostik, Ausgangbefund für Verlaufsbeurteilung	*Wenn initial erhöht:* ACE, löslicher IL-2-Rezeptor, Neopterin	Verlaufsbeurteilung

Speziellere Laborbiomarker wie „angiotensin-converting enzyme“ (ACE), löslicher Interleukin-2-Rezeptor oder Neopterin sind zwar alleine in der Diagnostik unspezifisch, können aber in unklaren Situationen hilfreich sein und – vor allem wenn initial erhöht – als zusätzliche Verlaufsparameter dienen [12, 13].

### Radiologische Diagnostik

Die initiale Evaluierung erfolgt in der Regel mittels Thorax-Röntgen, dessen Sensitivität jedoch in frühen oder subklinischen Krankheitsstadien limitiert ist. Die pulmonale Sarkoidose wird historisch thoraxradiographisch in Stadien nach Scadding eingeteilt: Stadium I und II umfassen eine mediastinale/hiläre Lymphadenopathie (LAP) mit/ohne Lungenparenchymbefall, Stadium III eine Lungenparenchymbeteiligung ohne begleitende Lymphadenopathie und das Stadium IV die Ausbildung einer Lungenfibrose („sarcoidosis-associated pulmonary fibrosis“ [SAPF]) [1, 2, 15, 16].

**Abb. 2:**
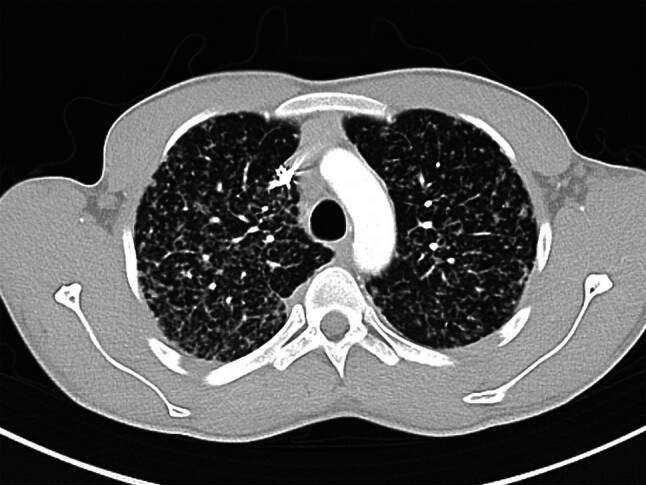
Lungensarkoidose mit multiplen Mikronoduli mit perilymphatischer Anordnung (unter Einbeziehung von Pleura und Fissuren). (Quelle: KUK Linz, Universitätsklinik für Innere Medizin 4 – Pneumologie)

Die Computertomographie (CT) des Thorax ist ein wesentlich präziseres diagnostisches Werkzeug, welches eine detaillierte Darstellung granulomatöser Manifestationen sowie lymphatischer Involvierung intra- und extrathorakal ermöglicht (Abb. 2 und 3).

**Abb. 3:**
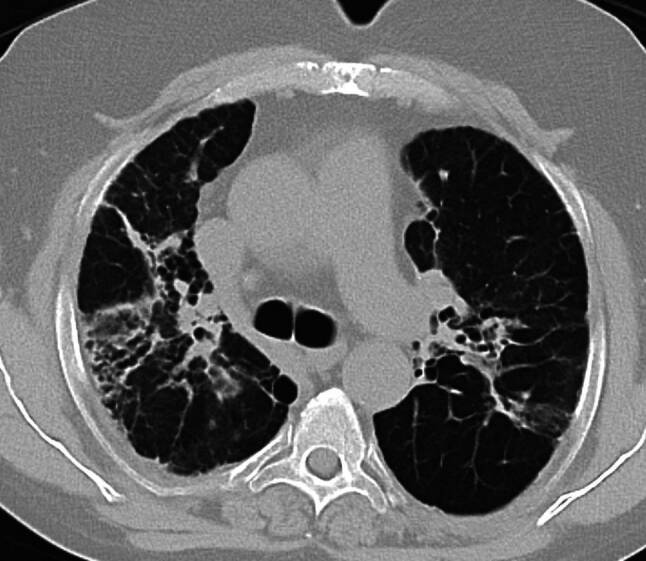
CT-morphologisches Bild einer SAPF mit bronchozentrischer Betonung, Bildung von Traktionsbronchiektasen und Volumsreduktion. (Quelle: KUK Linz, Universitätsklinik für Innere Medizin 4 – Pneumologie)

In der CT werden Lungenparenchymveränderungen bei Sarkoidose in nichtfibrotische und fibrotische Veränderungen unterteilt, wobei nichtfibrotische Lungenparenchymveränderungen sich als multiple peribronchovaskuläre, perifissurale oder subpleurale Mikronoduli, als multiple größere peribronchovaskuläre Noduli, als verstreute größere Knötchen oder als Konsolidierung als die vorherrschende oder einzige Anomalie manifestieren [17]. Eine Lungenfibrose bei Sarkoidose kann sich mit bronchozentrischen Retikulationen mit oder ohne dichte parenchymale Konsolidierungen, mit oder ohne Kavernenbildung oder als große bronchozentrische Konsolidierungen manifestieren [17]. Vor allem eine bihiläre Lymphadenopathie in Kombination mit mikronodulären Parenchymveränderungen mit perilymphatischer Anordnung (Beteiligung der Pleura und der Fissuren) ist bei entsprechendem klinischem Kontext hochgradig suggestiv für die pulmonale Sarkoidose [18].

### ^18^F-FDG-PET/CT und andere spezielle Bildgebung

Die ^18^Fluor-Fluordesoxyglucose-Positronenemissionstomographie in Kombination mit der CT (18F-FDG-PET/CT) gewinnt in speziellen Indikationen bei Sarkoidose an Bedeutung [3]. Die aktuelle Leitlinie der American Thoracic Society (ATS) zur Diagnostik der Sarkoidose empfiehlt die Durchführung einer nach Fasting-Protokoll vorbereiteten PET/CT bei Patienten mit suspizierter Herzbeteiligung, wenn eine Herz-Magnetresonanztomographie (MRT) nicht möglich oder inkonklusiv ist [8]. In Fällen von pulmonaler Sarkoidose ermöglicht die 18F-FDG-PET/CT eine hochsensitive Darstellung der Entzündungsaktivität der Lunge, der mediastinalen Lymphknoten sowie der extrathorakalen Beteiligung inklusive Identifikation möglicher leicht zugänglicher Biopsiestellen. Außerdem kann insbesondere bei Multiorgansarkoidose und in therapierefraktären Fällen das Therapieansprechen beurteilt werden [11, 19, 20].

Bei speziellen Fragestellungen kommt je nach Organbefall auch die MRT zum Einsatz, z. B. in der Abklärung der kardialen Sarkoidose, wobei das Auftreten eines Late-Gadolinium-Enhancements (LGE) das Vorhandensein fibrotischer sowie entzündlicher Prozesse im Myokard signalisiert [21].

## Pathogenese

Die granulomatöse Entzündung der Sarkoidose wird als dysregulierte Immunreaktion auf noch unbekannte Antigene aus der Umwelt bei genetisch anfälligen Personen angesehen [4, 9]. Ein erhöhtes Risiko für Sarkoidose wurde bei Menschen mit Exposition gegenüber Insektiziden, Schimmelpilzen, Metall-, anorganischen und organischen Stäuben sowie in Berufen wie der Brandbekämpfung und der Landwirtschaft identifiziert [4, 22]. Mikroben wie *Cutibacterium acnes* und verschiedene Mykobakterienarten wurden ebenfalls mit der Krankheit in Verbindung gebracht [4].

Bei positiver Familienanamnese besteht ein 2‑ bis 4faches Risiko, die Krankheit zu entwickeln [23, 24]. Varianten von Genen, die an der Antigenpräsentation beteiligt sind (HLA-Klasse II), und andere Gene wie BTNL2 und Tumor-Nekrose-Faktor‑α (TNF-α) wurden mit Sarkoidose assoziiert [4].

Diese Dysregulationen in der angeborenen Immunantwort können zur Persistenz von epitheloidzelligen Granulomen führen, die dann als Brennpunkt für weitere Inflammation und Entwicklung von Fibrose fungieren können [4]. Der „mammalian target of rapamycin complex“(mTORC1)-Stoffwechselweg hält beispielsweise die Granulombildung aufrecht [25–28]. Die Ansammlung von CD4-T-Zellen in den betroffenen Organen ist ein weiteres Kennzeichen der Sarkoidose, und Zytokine wie TNF‑α, Interferon‑γ (IFN-γ), IL‑6 und transformierender Wachstumsfaktor (TGF-β) werden ebenfalls hochreguliert [3, 4, 12].

## Allgemeine Therapieziele

Sarkoidose präsentiert sich in unterschiedlichsten Ausprägungen und Verlaufsformen – vom asymptomatischen Zufallsbefund, dem akuten, oft selbstlimitierenden Löfgren-Syndrom bis hin zur chronischen Multiorganform und zum plötzlichen Herztod. Trotzdem hat sich eine einfache Regel etabliert: Behandeln, um Organschäden zu vermeiden oder um die Lebensqualität zu verbessern [29].

Die Einschätzung der Organgefährdung erfordert einerseits klinische Untersuchungen, andererseits auch die Befragung des Patienten, speziell im Hinblick auf kardiale Symptome (z. B. Synkopen, Palpitationen). Die Lebensqualität wird häufig auch durch nicht organspezifische Symptome wie Fatigue beeinflusst [30].

Ein strukturierter Untersuchungsplan und ein definiertes Behandlungsziel sind für die gemeinsame Entscheidung zur Therapie hilfreich [4]. Während sich granulombedingte Probleme meist gut medikamentös behandeln lassen, beeinträchtigen Fatigue sowie Gelenk- und Muskelprobleme selbst gut therapierte Patienten oft noch beträchtlich [31]. Naheliegenderweise werden deshalb in Befragungen von Patienten das Funktionieren im Alltag und Lebensqualität als wichtigste Therapieziele genannt [32]. Problematisch wird von Patienten auch das Thema der oft nötigen, aber nebenwirkungsträchtigen Steroidtherapie wahrgenommen.

Der subjektive Begriff Lebensqualität kann über sog. PROMs („patient-reported outcome measures“) objektiviert werden. Hierfür existieren verschiedene Fragebögen, wie z. B. der King’s Sarcoidosis Questionnaire [33], welche allerdings eher in Studiensettings als in der täglichen Praxis Anwendung finden. Für die Klinik gilt aber jedenfalls, dass nicht nur organspezifische Symptome abgefragt werden sollen, sondern auch nichtorganbezogene Symptome wie Fatigue, Schmerzen, alltägliche kognitive Störungen, Small-fiber-Neuropathie, Bewegungseinschränkung und Depressionen [34]. Die Wertschätzung der patientenberichteten Symptome ist wichtig: So wird schwere Dyspnoe im Vergleich selten von Lungenpatienten, aber häufiger von Patienten mit kardiologischer Beteiligung berichtet [35]. Fatigue bleibt bei 2 von 3 Patienten auch nach Verschwinden aller organspezifischen Symptome bestehen. Begleitende Gedächtnisprobleme und depressive Symptome prognostizieren Fatigue, eine Lungenbeteiligung per se tut es nicht [36].

Ein kürzlich vorgestelltes Betreuungsmodell umfasst 5 Stufen [37]:

- die Beurteilung der Symptome und Bedürfnisse des Patienten,
- die Stärkung des Patienten durch Unterstützung und Aufklärung,
- die Behandlung von Beschwerden und Komorbiditäten,
- die medikamentöse Behandlung der Organmanifestationen,
- die Behandlung und Einbindung extrapulmonaler Spezialisten bei Bedarf.

Die Aufklärung und Schulung sind die Voraussetzung für eine qualifizierte Mitentscheidung des Patienten zur Therapie, aber auch eine notwendige Qualifikation zur Darstellung und Diskussion von Symptomen und ein Mittel zur Verbesserung der Lebensqualität. Auf dieser Basis soll das Behandlungsziel, individuell an Klinik und Risikokonstellation angepasst, zwischen Patient und Behandler definiert werden.

## Medikamentöse Therapie

**Abb. 4:**
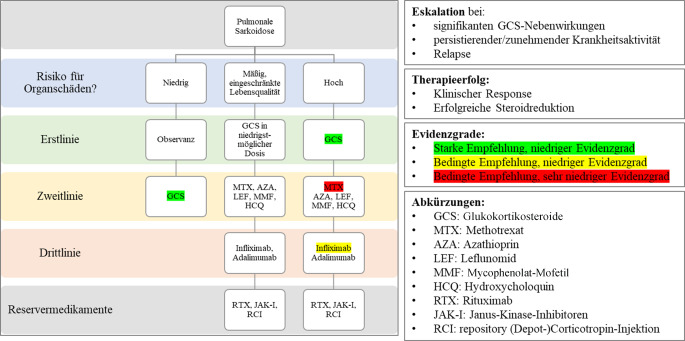
Therapiealgorithmus am Beispiel der pulmonalen Sarkoidose. (Nach Baughman RP et al. [40])

Die Indikation zur Behandlung hängt von 2 Hauptfaktoren ab: (1) Risiko für Tod oder Organschaden und (2) Beeinträchtigung der Lebensqualität durch Symptome [38]. Die Gesamtmortalität der Sarkoidose liegt bei etwa 5 % [39–41]. Die kardiale sowie pulmonale Beteiligung inklusive pulmonaler Hypertonie und Lungenfibrose stellen die häufigsten Todesursachen dar [4, 40, 42–45]. Die aktuell empfohlene Therapie – unabhängig vom Manifestationsort – versteht sich als Stufentherapie (Abb. 4), beginnend mit Glukokortikoiden als „first-line“ [40].

## Erstlinientherapie

### Glukokortikoide

Dosis: Prednison/Äquivalent 20–40 mg/Tag, in absteigender Dosierung.

Primäres Therapeutikum der Wahl ist ein Glukokortikoid in der niedrigsten möglichen Dosierung und Therapiedauer. Eine Langzeitanwendung ist mit erheblicher Organ- und Systemtoxizität verbunden [13, 40, 46, 47]. Daher empfiehlt ein rezenter Delphi-Konsens eine Begrenzung der anfänglichen Kortikosteroidtherapie auf 3 bis 6 Monate mit dem Ziel, nach klinischem Ansprechen auf eine Erhaltungsdosis von < 10 mg Prednison/Tag zu reduzieren [48]. Im Falle einer Langzeitkortisontherapie (> 3 Monate) werden eine ausführliche Aufklärung über das Nebenwirkungsprofil und eine Osteoporoseabklärung empfohlen [49]. Bei Normokalzämie sollte entsprechend den allgemeinen Empfehlungen auf eine ausreichende Kalzium- (1000–1200 mg/Tag) und Vitamin-D-Zufuhr (Serum-Vitamin-D3-Ziel ≥ 30–50 ng/ml) geachtet werden [49], bei Mangelzuständen soll unter regelmäßiger Laborkontrolle substituiert werden. Eine Kalziumsubstitution bei Sarkoidose sollte immer unter regelmäßigen Laborkontrollen erfolgen.

## Zweitlinienbehandlung bei Nichtansprechen auf Kortikosteroide bzw. als glukokortikoidsparende Medikation

In symptomatischen Fällen, in denen die Krankheit trotz Kortikosteroidtherapie fortbesteht, inakzeptable Steroidnebenwirkungen auftreten oder ein hohes Risiko für Mortalität oder bleibende Organschäden besteht, ist eine steroidsparende, nichtbiologische immunsuppressive Zweitlinientherapie indiziert [40].

### Methotrexat (MTX)

Dosierung: 10–15 mg wöchentlich, dazu Folsäure 5 mg 1‑ bis 2‑mal wöchentlich [40].

Die Dosis von Methotrexat (MTX) kann bei Bedarf und akzeptablem Nebenwirkungsprofil auf 20 mg oder mehr erhöht werden. Es wird empfohlen, MTX mit Folsäure zu kombinieren [40, 50]. Subkutanes MTX wird bei ungenügendem klinischem Ansprechen und/oder gastrointestinalen Nebenwirkungen empfohlen [51]. Verlässliche Empfängnisverhütung ist angezeigt!

### Azathioprin (AZA)

Dosierung: 50–250 mg täglich [40].

Eine genetische Analyse der TPMT-Allele vor Einleitung kann helfen, die Verträglichkeit vorherzusagen, Mittel der Wahl bei Kinderwunsch.

## Drittlinientherapie in refraktären Fällen (Auswahl)

Es besteht eine Vielzahl an Drittlinientherapeutika, wobei TNF-α-Inhibitoren die gängigsten Medikamente sind. Eine Auswahl ist hier genannt, für detaillierte Informationen verweisen wir auf die jeweiligen Fachgesellschaften und Leitlinien [40].

*Infliximab:* Dosis 3–5 mg/kg i.v. initial und nach 2 Wochen; Wiederholung dann alle 4 bis 6 Wochen [40, 52].

*Adalimumab:* Dosis 40 mg alle 1 bis 2 Wochen s.c. [40, 53].

## Antifibrotische Therapie

*Nintedanib:* Im Falle eines progressiv fibrosierenden Verlaufs einer SAPF soll eine antifibrotische Medikation mit Nintedanib erfolgen [54, 55].

## Zukünftige Entwicklungen: neue therapeutische Ansätze

Neue Erkenntnisse betreffen den JAK-STAT-Signalweg und die Typ-1-Immunität. Die Wirksamkeit von Tofacitinib (5–10 mg 2‑mal täglich) bei Patienten mit kutaner Sarkoidose ist umfassend nachgewiesen, wenn auch nur in kleinen Kohorten [56–58].

In einer Reihe von Studien wurde eine aktive mTOR-Signalübertragung in Granulomen von Sarkoidosepatienten mit Lungen- [25, 27], Herz- [28, 59] und Hautbefall [60] nachgewiesen. Klinische Studien mit dem systemischen mTOR-Inhibitor Sirolimus haben eine Wirksamkeit bei Patienten mit kutaner Sarkoidose [61] (6 mg 1‑malig und 2 mg oral/Tag über 4 Monate) mit einer lang anhaltenden Wirkung von mehr als einem Jahr nach der Behandlung und bei einem Lungensarkoidosepatienten [62] (2 mg/Tag/10 Monate) mit einer Besserung der CT und der Hustensymptomatik gezeigt.

Für alle hier genannten Substanzen gilt, dass noch weitere, größere klinische Studien nötig sind, bis sie als Routinetherapie für Sarkoidose empfohlen werden können.

## Pulmonale Sarkoidose

Die pulmonale Beteiligung stellt mit bis zu 95 % die häufigste Manifestation der Sarkoidose dar und ist oft durch eine mediastinale und bihiläre LAP charakterisiert. Eine Parenchymbeteiligung kann sich als typisch noduläres Muster mit perilymphatischer Verteilung bis hin zur irreversiblen Fibrose („sarcoidosis-associated pulmonary fibrosis“ [SAPF]) manifestieren [1, 2, 16, 63]. Mit zunehmendem Parenchymbefall nehmen die Spontanremissionsrate und die generelle Prognose ab [4, 15, 44].

Die klinische Präsentation ist sehr variabel und bei Erstmanifestation teils oligo- bis asymptomatisch. Respiratorische Symptome wie Husten, Dyspnoe oder thorakales Druckgefühl, systemische Zeichen wie Fieber, B‑Symptomatik, Fatigue oder Symptome anderer Organmanifestationen können vorliegen [44].

Die thorakale Beteiligung bei akuten Formen der Sarkoidose (beispielsweise Löfgren-Syndrom oder Heerfordt-Syndrom) weist mit bis zu 85 % eine hohe Rate an Spontanremissionen auf [4, 44]. Eine invasive Diagnostik ist hier in aller Regel bei typischer Klinik und Bildgebung nicht indiziert, jedoch eine engmaschige Verlaufskontrolle. Die Therapie besteht primär in einer symptomorientierten Behandlung, wie beispielsweise mit NSAR [4, 8, 40].

Je nach Befallsmuster kann die Lungenfunktion unauffällig, obstruktiv oder restriktiv sein, sowie eine Diffusionsstörung aufweisen. Belastungsuntersuchungen wie der 6‑Minuten-Gehtest oder die Spiroergometrie können funktionell relevante pulmonale Beteiligungen demaskieren [64].

Der Entscheidung zur histologischen Diagnosesicherung sollte eine Risiko-Nutzen-Abwägung sowie die Abschätzung der Wahrscheinlichkeit benigner und maligner Differenzialdiagnosen (z. B. Lymphom, Bronchialkarzinom, Tuberkulose, Silikose) vorausgehen. Eine reine LAP mit typischer Präsentation und ohne Therapieindikation kann durchaus ohne histologische Sicherung beobachtet werden, wobei eine Abwägung im Einzelfall erfolgen soll [8]. Wenn eine invasive Abklärung notwendig erscheint, sollte diese bei LAP mittels endobronchialer ultraschallgezielter Lymphknotenbiopsie (EBUS-TBNA) erfolgen, eine lymphozytäre BAL mit CD4/CD8-Ratio > 3,5 kann die Diagnose unterstützen. Bei makroskopisch auffälliger Bronchialschleimhaut ist eine Zangenbiopsie empfohlen, Biopsien aus dem Lungenparenchym sind insgesamt nur selten nötig [8, 44].

Die Therapie von potenziell chronischen Formen der Sarkoidose abseits von akuter Organ- oder Lebensbedrohung sollte im Rahmen von „shared decision making“ mit den Patienten gemeinsam getroffen werden und ist von unterschiedlichen Faktoren abhängig: Für die Einleitung einer Therapie sprechen eine Einschränkung der Lebensqualität aufgrund von Symptomen sowie das Risiko einer Organschädigung z. B. bei Verlust von Lungenfunktion, ausgedehntem Lungenparenchymbefall und/oder Ausbildung von fibrotischen Veränderungen [40].

## Kutane Sarkoidose

Bis zu 30 % der Sarkoidosepatienten präsentieren sich mit einer Hautmanifestation, oft als Erstsymptom [65].

Bei Hautsarkoidose wird einerseits zwischen akut und chronisch, andererseits zwischen spezifischen und unspezifischen Hautmanifestationen differenziert (Tab. 2; [66–68]). Dies ist wichtig, da die Diagnose einer Hautsarkoidose nur bei spezifischen Hautmanifestationen aus der Hautbiopsie gesichert werden kann. Aufgrund der vielen unterschiedlichen Ausprägungen der Hautsarkoidose sollte ein Facharzt für Dermatologie zur Beurteilung herangezogen werden.

**Tab. 2:** Unterschiedliche Hautmanifestationen der Hautsarkoidose [66–68]

Häufigkeit	Spezifische Hautmanifestationen	Unspezifische Hautmanifestationen
Sehr häufig	Papeln	Erythema nodosum
	Plaques	–
Häufig	Lupus pernio	Steroidakne
	Subkutane Knoten	–
	Narbensarkoidose	–
Selten	Ichthyosiform	Calcinosis cutis
	Nagelbeteiligung	Trommelschlegelfinger
	Alopezie	Psoriasis
	Erythrodermie	–
	Ulzera	–

Das Erythema nodosum ist eine unspezifische Hautmanifestation der akuten Verlaufsform der Sarkoidose, dem Löfgren-Syndrom und präsentiert sich mit livid-roten Knoten am häufigsten im Bereich der Schienbeine. Histologisch zeigen sich eine septale Pannikulitis, aber keine nackten Granulome. Die Diagnose eines Löfgren-Syndroms wird klinisch in Zusammenschau mit der weiteren Organsymptomatik (Erythema nodosum, bihiläre Lymphadenopathie, Arthritis) gestellt.

**Abb. 5:**
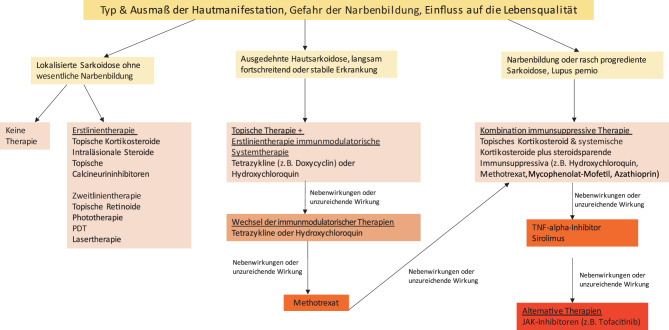
Therapiealgorithmus der Hautsarkoidose. (Nach Caplan A et al. [66])

Die Therapie der Hautsarkoidose wird bestimmt durch den Typ der Hautmanifestation, die Ausprägung, die Gefahr einer Narbenbildung, den Leidensdruck des Patienten und das Ausmaß der weiteren Organbeteiligung (Abb. 5). Die Behandlung der Haut (z. B. durch topische Therapien) muss auch mitbedacht werden, wenn primär andere Organsysteme das Therapieregime bestimmen. Wird eine Systemtherapie begonnen, sollte diese für mindestens 3 Monate durchgeführt werden, bevor die Wirksamkeit bezüglich der Haut bewertet wird [61, 66–68].

## Artikuläre und muskuläre Manifestationen

Muskuloskeletale Manifestationen treten bei bis zu einem Drittel der Patienten auf, können komplexe Krankheitsbilder bedingen und reichen von Arthralgien bis zu destruktiven ossären Läsionen [69, 70]. Muskuloskeletale Beteiligungen zeigen sich in der Regel früh im Verlauf der Erkrankung. Verschiedene bildgebende Verfahren wie die 18F-FDG-PET/CT könnten sich als nützlich erweisen, um das Ausmaß der Beteiligung und die Aktivität der Krankheit zu erfassen [71]. Bei der Behandlung der rheumatischen Sarkoidosemanifestationen haben bis dato Glukokortikoide einen zentralen Stellenwert inne. Methotrexat stellt das bevorzugte steroidsparende Agens dar, sofern keine renale Beteiligung oder andere Kontraindikationen vorliegen. Therapien mit Biologika, insbesondere TNF-α-Inhibitoren, werden in schwerwiegenden Fällen eingesetzt [70, 72].

### Gelenkbeteiligung

#### Akute Arthritis (Löfgren-Syndrom)

Die häufigste muskuloskeletale Manifestation der Sarkoidose ist das Löfgren-Syndrom, welches die Triade aus symmetrischer hilärer Lymphadenopathie, (Peri‑)Arthritis und Erythema nodosum umfasst [73]. Die Gelenkbeteiligung betrifft typischerweise beide Sprunggelenke, kann selten aber auch andere Regionen einschließlich der Knie‑, Hand‑, Ellenbogen- und Metakarpophalangealgelenke (MCP) betreffen [74]. Sonographisch imponiert die betroffene Gelenkregion meist durch eine periartikuläre Weichteilschwellung und Tenosynovitis. Die Erkrankung tritt saisonal gehäuft insbesondere im Frühling auf und zeichnet sich durch eine vergleichsweise hohe Spontanremissionsrate und niedrige Rezidivwahrscheinlichkeit aus [75, 76]. Bei entsprechender klinischer Präsentation ist eine histologische Gewebssicherung in der Regel nicht notwendig [70], die Behandlung erfolgt symptomatisch mittels nichtsteroidaler Antirheumatika (NSARs).

#### Chronische Arthritis

Die chronische Form der Sarkoidose-assoziierten Arthritis geht oftmals mit anderen extrapulmonalen Manifestationen, insbesondere der Hautbeteiligung, einher [77]. Charakteristisch ist eine symmetrische Oligoarthritis der mittleren bis großen Gelenke. Destruktiv verlaufende Arthritiden sind selten. Das klinische Bild der Jaccoud-Arthropathie, die sich durch eine deformierende, jedoch nicht erosive Arthritis auszeichnet, kann beispielsweise bei Patienten mit Organbeteiligung im fortgeschrittenen Krankheitsstadium beobachtet werden [71]. Die Ausschlussdiagnose von Gicht, Kalziumpyrophosphatarthropathie (Pseudogicht) und septischer Arthritis ist wichtig. Eine Synovialbiopsie ist oftmals hilfreich, um ein granulomatöses Infiltrat nachzuweisen und andere Differenzialdiagnosen auszuschließen [70]. Eine axiale, die Wirbelsäule respektive das Sakroiliakalgelenk affektierende Verlaufsform ist oft asymptomatisch – Läsionen werden oft während bildgebender Untersuchungen per Zufall detektiert [78]. Obwohl die Sakroiliitis im Rahmen der Sarkoidose in der Regel unilateral auftritt, kann es ohne bioptische Abklärung schwierig sein, sie von Tuberkulose oder einer durch eine andere Infektion verursachten Sakroiliitis zu differenzieren. Eine weiterführende Abklärung und die Bestimmung von HLA-B27 kann hilfreich sein, um sie von einer axialen Spondyloarthritis (SpA) zu unterscheiden [71, 79].

#### Daktylitis

Eine weitere Manifestationsform der Sarkoidose-assoziierten Arthropathie ist die Daktylitis, welche sonst typischerweise bei Psoriasisarthritis auftritt und durch plumpe, aufgetriebene, verdickte und gerötete „Wurstfinger“ charakterisiert ist [70]. Sie ist gehäuft bei Patienten afrikanischer Abstammung und in Zusammenhang mit einer systemischen Beteiligung [70, 71], typischerweise asymmetrisch an den zweiten und dritten Fingergliedern unter Aussparung der MCP-Gelenke lokalisiert [80]. Histologisch finden sich Tenosynovitis und Granulome im Weichgewebe [81].

### Muskelbeteiligung

Die Sarkoidose-assoziierte Myopathie mit Beteiligung der Skelettmuskulatur tritt histologisch in bis zur Hälfte aller Sarkoidosepatienten auf, jedoch hat nur ein Bruchteil (0,5–2 %) eine klassische Symptomatik [70, 82, 83]. Das Beschwerdebild umfasst neben generalisierter Schwäche, Fatigue und reduzierter Leistungsfähigkeit auch Myalgien sowie eine proximal betonte Muskelschwäche [84]. Die Unterscheidung zu anderen Muskelerkrankungen kann oftmals herausfordernd sein [85], insbesondere bei Myopathie unter Glukokortikoidtherapie. Eine Muskelbiopsie ist hier zur Differenzialdiagnose hilfreich [70].

## Okuläre Sarkoidose

Die Sarkoidose kann jede Struktur von Auge und Augenanhangsgebilden wie Orbita, Lider, Tränendrüse und -wege, Bindehaut und das Augeninnere betreffen. Der Anteil der okulären Beteiligung bei systemischer Sarkoidose wird in der Literatur mit 10–71 % recht variabel angegeben [86]. Die häufigste visusrelevante Manifestation ist eine Entzündung der Uvea, der mittleren Augenhaut. Die Uveitis wird nach dem primären Fokus der Entzündung in Uveitis anterior (Iris, Ziliarkörper), intermedia (Vitreus, Pars plana), posterior (Choroidea, Retina) und Panuveitis (alle Bereiche betroffen) unterteilt. Uveitis ist also ein Überbegriff für klinisch und prognostisch unterschiedliche Krankheitsbilder. Eine Uveitis wird bei 20–30 % aller Patienten mit Sarkoidose beschrieben. Häufig ist die Uveitis dabei die klinische Erstmanifestation einer systemischen Sarkoidose (30–79 %) [87]. Bei mehr als einem Drittel der Sarkoidose-assoziierten Uveitisfälle bleibt die Uveitis die einzige Manifestation einer Sarkoidose.

Die Diagnose einer okulären Sarkoidose kann insbesondere in Abwesenheit systemischer Zeichen schwierig sein. Eine Biopsie von intraokulärem Gewebe wird aufgrund des Komplikationsrisikos nur selten in dieser Indikation durchgeführt. Diagnostische Kriterien wurden vom IWOS (International Workshop on Ocular Sarcoidosis) erstellt [88]. Diese umfassen 7 typische klinische intraokuläre Zeichen (z. B. Irisknötchen, noduläre oder segmentale Periphlebitis) sowie diverse systemische Untersuchungen, die zum Verdacht einer okulären Sarkoidose führen sollen. Die Symptome einer Uveitis bei Sarkoidose sind allesamt unspezifisch und je nach Uveitisform, Lokalisation und zeitlichem Verlauf unterschiedlich. Photophobie, Skotome, Schleiersehen sowie Schmerzen und Rötungen sind häufigere Symptome.

Die Therapie der Sarkoidose-assoziierten Uveitis ist abhängig von Schweregrad und Uveitisform. Kortikosteroide sind der Hauptpfeiler der Behandlung. Da topisches Kortison nur im vorderen Augensegment wirkt, ist in der Regel eine systemische Therapie angezeigt. Lokale Therapiealternativen sind periokuläre und intravitreale Kortisoninjektionen, deren Nutzen/Risiko im Einzelfall abzuwägen ist. Bei chronischem Verlauf sowie häufigen Rezidiven besteht die Indikation für eine systemische immunsuppressive Therapie (Antimetabolite, Calcineurinantagonisten) oder Biologika, wobei eine Zulassung für die Behandlung einer Uveitis derzeit nur für Adalimumab besteht [89].

Bei Verdacht und zum Ausschluss einer aktiven Entzündung genügen augenärztliche Standarduntersuchungen. Eine zeitnahe Überweisung zum Augenfacharzt sollte bei unklaren akuten oder rezidivierenden Augenrötungen und -schmerzen erfolgen oder bei unklaren Sehstörungen. Die Betreuung an einem Spezialzentrum für Uveitis ist angezeigt bei schweren Uveitisfällen und chronischen oder häufig rezidivierenden Verläufen.

## Pulmonale Hypertonie

Anhand der aktuellen ESC/ERS pulmonale Hypertonie(PH)-Leitlinien wird eine PH durch den Anstieg des pulmonalarteriellen Mitteldrucks (mPAP) > 20 mm Hg definiert [90, 91]. Wenn eine PH bei einer Sarkoidose auftritt, wird diese laut der aktuellen klinischen Klassifikation in die Gruppe 5 (PH mit unklaren und/oder multifaktoriellen Mechanismen) eingeteilt. Epidemiologische Studien berichten, dass 6–20 % der Patienten mit Sarkoidose eine PH entwickeln [92]. Die Ursache der pulmonalen Druckerhöhung ist multifaktoriell, wobei die Bildung von Granulomen in den Lungengefäßen, entzündliche Veränderungen, chronische Thromboembolien, fibrosierende Prozesse in der Lunge und im Mediastinum sowie die Kompression der Lungengefäße durch Lymphknoten eine Rolle spielen können.

Das häufigste Symptom einer PH bei Sarkoidose ist eine Belastungsdyspnoe, welche nicht mit der Grunderkrankung selbst erklärt werden kann. Die wichtigste nichtinvasive Untersuchungsmethode ist die Echokardiographie, welche direkte und indirekte Hinweise für eine pulmonale Druckerhöhung liefert. Die Diagnose wird mit einer Rechtsherzkatheteruntersuchung bestätigt, welche die Messung des mPAP und die Berechnung des pulmonalen Gefäßwiderstandes erlaubt [90, 91].

Es liegen derzeit keine zugelassenen Medikamente zur spezifischen Therapie der PH bei Sarkoidose vor. Kleinere Studien zeigten eine hämodynamische bzw. klinische Besserung auf zugelassene PH-Medikamente, diese Ergebnisse sind aber nicht in größeren Untersuchungen validiert [93–96]. Bei entzündlich aktiver Erkrankung wurde auch über die Effektivität einer Behandlung mit Kortikosteroiden bzw. immunsuppressiven Medikamenten berichtet. Die Prognose einer schweren PH bei Sarkoidose ist schlecht, deswegen soll in ausgewählten Fällen rechtzeitig eine Lungentransplantation in Erwägung gezogen werden [97].

## Kardiale Sarkoidose

Eine kardiale Beteiligung tritt bei 25 % aller Patienten mit Sarkoidose auf, allerdings ist sie nur in 2–7 % klinisch relevant [98]. In diesen Fällen stellt die kardiale Beteiligung jedoch einen schwerwiegenden Befund dar, da 13–25 % aller Sarkoidose-bedingten Todesfälle auf die kardiale Mitbeteiligung zurückgeführt werden [3, 99]. Ungefähr bei einem Drittel der Patienten mit kardialer Sarkoidose besteht eine isolierte kardiale Beteiligung. Die kardiale Sarkoidose ist pathophysiologisch durch eine inflammatorische, potenziell reversible granulomatöse Infiltration, gefolgt von einer irreversiblen Fibrosierung charakterisiert. Der direkte histologische Nachweis mittels Endomyokardbiopsie gelingt aufgrund der fokalen Infiltration nur in ca. 25 % der Fälle („sampling error“) [100–102].

Je nach Lokalisation und Ausmaß der Infiltrate treten Erregungsleitungsstörungen, ventrikuläre Arrhythmien und eine abnehmende systolische Funktion auf. Besonders bei Patienten im Alter unter 60 Jahre mit hochgradigem AV-Block, ventrikulären Arrhythmien bis hin zum plötzlichen Herztod oder nichtischämischer Herzinsuffizienz ist die kardiale Sarkoidose daher eine wichtige Differenzialdiagnose. „Red flags“ sind neben Palpitationen, Synkopen und Zeichen bzw. Symptomen der Herzinsuffizienz regionale, nicht durch KHK erklärbare Wandbewegungsstörungen mit narbiger Wandverdünnung im Herzultraschall (v. a. basal inferiores Septum), des Weiteren treten häufig Erregungsleitungsstörungen wie Schenkel- oder AV-Block im EKG auf [103]. Bei gesicherter extrakardialer Sarkoidose ist dementsprechend eine Anamnese hinsichtlich kardialer Symptome empfohlen, ebenso wie die Durchführung eines Routine-EKG. Eine Echokardiographie kann weiterführend erwogen werden. Die oben genannten „red flags“ sind indikativ für das Vorliegen einer kardialen Beteiligung und sollten ein Screening auf kardiale Beteiligung nach sich ziehen [99, 104].

Bei einer isolierten kardialen Sarkoidose ist gemäß internationalen Richtlinien ein bioptischer Nachweis gefordert, welcher aufgrund der niedrigen Sensitivität der Endomyokardbiopsie jedoch häufig nicht gelingt [8, 98]. Diagnostisch wegweisend ist der Nachweis von fokaler Inflammation bzw. fibrotischer Areale in der kardialen MRT oder im Fasting-FDG-PET. Beim Fasting-PET wird ein spezifisches Patientenvorbereitungsprotokoll vor der Untersuchung eingehalten (12–24 h fettreiche Diät ohne Kohlenhydrate, 12–18 h Fasten und ggf. zusätzlich noch i.v. Heparin 15 min vor der Untersuchung) [105]. In der klinischen Realität wird daher häufig rein auf der Grundlage der nichtinvasiven Bildgebung im Kontext mit typischer Anamnese, Klinik und Zeichen einer kardialen Sarkoidose eine immunsuppressive Therapie eingeleitet, auch wenn ein bioptischer Sarkoidosebeweis nicht gelingt [106, 107]. Bei bioptisch gesicherter extrakardialer Sarkoidose ist die kardiale Bildgebung ausreichend für den Nachweis einer kardialen Mitbeteiligung [98]. Eine immunsuppressive Therapie ist indiziert bei Vorliegen eines AV-Blocks, ventrikulären Arrhythmien oder Herzinsuffizienz. Des Weiteren bestehen spezifische Indikationskriterien für die primärprophylaktische Implantation eines implantierbaren Kardioverter-Defibrillators (ICD) [104].

## Renale Sarkoidose

Die exakte Prävalenz der renalen Beteiligung der Sarkoidose liegt nach Schätzungen bei 10–30 % [108, 109]. Dabei wird grundlegend unterschieden zwischen:

- der renalen Sarkoidosebeteiligung im Sinne einer granulomatösen interstitiellen Nephritis,
- der durch Hyperkalzämie/-kalzurie verursachten Nephrokalzinose mit oder ohne Nephrolithiasis.

Hinsichtlich des Verlaufs treten sowohl akute Nierenfunktionseinschränkungen (AKI) als auch chronische Formen (CKD) auf.

Diagnostisch sollten bei jedem Sarkoidosepatienten eine Bestimmung des Serumkreatinins sowie eine Berechnung der glomerulären Filtrationsrate (GFR) erfolgen, um die quantitative Einschränkung der Nierenfunktion einschätzen zu können [8]. Des Weiteren sollten das Serumkalzium sowie – bei Auffälligkeiten in der Routinediagnostik – idealerweise das Parathormon und 1,25-Dihydroxy-Vitamin D_3_ (Calcitriol) bestimmt werden. Differenzialdiagnostisch kommen zahlreiche andere, häufigere Ursachen für ein AKI bzw. eine CKD infrage, die renale Sarkoidosebeteiligung ist daher meist eine Ausschlussdiagnose. Bildgebend sollte eine Sonographie der Nieren und ableitenden Harnwege bei jedem Verdacht auf Nierenbeteiligung durchgeführt werden [110].

Die Nephrokalzinose ist die häufigste renale Komplikation einer Sarkoidose und Ausdruck einer sekundären Schädigung durch eine Hyperkalzurie und verläuft häufig asymptomatisch. Bei Vorliegen einer Nephrolithiasis oder Hyperkalzämie sollte unbedingt eine suffiziente medikamentöse Sarkoidosetherapie erfolgen, zusätzlich empfiehlt sich ein konventionelles CKD-Management [111].

Stellt sich der Verdacht auf eine interstitielle Beteiligung im Sinne einer granulomatösen Nephritis (mittelgradig bis stark eingeschränkte Nierenfunktion mit AKI III° oder CKD G4–5, häufig diffus aktive Sarkoidose, unauffälliges Harnsediment), sollte an eine Nierenbiopsie zur Diagnosesicherung gedacht werden [112, 113]. Hier zeigen sich nicht-verkäsende interstitielle Granulome, welche bei fehlendem bzw. geringem Fibrosierungsgrad als therapeutische Konsequenz eine Kortikosteroidtherapie bedingen [114].

## Neurosarkoidose

In 5–10 % kommt es zum Auftreten neurologischer Symptome im Rahmen einer Sarkoidose [115]. Darüber hinaus konnte bei 15–25 % eine asymptomatische nervale Beteiligung mittels histologischer Aufarbeitung in Autopsiestudien nachgewiesen werden [116]. Eine isolierte Neurosarkoidose tritt in rund 10–20 % auf. Diese Fälle stellen eine besondere diagnostische Herausforderung dar, da die Abgrenzung zu anderen neurologischen Erkrankungen schwierig ist [115].

Die Neurosarkoidose (NS) betrifft bevorzugt das zentrale Nervensystem inklusive Hirnnerven, kann jedoch seltener auch das periphere Nervensystem einschließlich der Muskulatur involvieren. Das Vorliegen einer aseptischen basalen Meningitis (in ca. 30 % der Fälle) sowie eine ein- oder beidseitige Hirnnervenaffektion (50–75 %) gelten als „typisches“ Bild einer Neurosarkoidose. Hierbei kommt es bevorzugt zu einer Beeinträchtigung des N. opticus (in ca. einem Drittel der Fälle) mit Auftreten einer Sehstörung oder einer peripheren Fazialisparese (in ca. einem Viertel der Fälle) [117]. Seltener manifestiert sich eine NS mit Ausfällen anderer Hirnnerven, einer Myelopathie (in ca. einem Viertel der Fälle), Polyneuropathie (zumeist in Form einer Mononeuritis multiplex, axonal führenden sensomotorischen Polyneuropathie oder Small-fiber-Polyneuropathie) oder Myopathie (< 10 %). Ausgeprägte leptomeningeale und parenchymatöse Veränderungen mit Involvierung der Hypophysenachse, Enzephalopathien, Hydrozephalus oder epileptischen Anfällen sind beschrieben [116].

Bei Verdacht auf eine neurologische Beteiligung sind zusätzlich zur allgemeinen Sarkoidoseabklärung eine Bildgebung mittels MRT und eine Lumbalpunktion indiziert. Eine pathognomonische Befundkonstellation der Bildgebung und des Liquors existiert allerdings nicht. Die MRT sollte mit Kontrastmittel (KM) durchgeführt werden und liefert in bis zu 80 % der Fälle mit NS einen auffälligen Befund wie eine leptomeningeale Anreicherung (65 %) oder basale und periventrikuläre Hyperintensitäten in T2-gewichteten Bildern (46 %) [118]. Der Liquor weist zumeist eine lymphozytäre Pleozytose mit fehlender intrathekaler Immunglobulinbildung auf, dennoch ist der Nachweis transienter oligoklonaler Banden möglich. Die zusätzliche Bestimmung von ACE, Beta-2-Mikroglobulin, Lysozym, löslichem IL-2-Rezeptor und Neopterin im Liquor können nützlich sein [119].

Neben der neurologischen Abklärung sollte eine intensive systemische Abklärung inklusive Ausschluss einer Tuberkulose erfolgen, auch um mögliche Biopsiestellen zu identifizieren, um nach Möglichkeit auf eine Nerven- oder Hirnbiopsie verzichten zu können. Ist das nicht möglich, muss eine solche aber dennoch in unklaren Fällen bei mutmaßlich isolierter NS aufgrund der Konsequenz einer langfristigen Immunsuppression ernsthaft erwogen werden.

Gesonderte wissenschaftlich abgesicherte Therapieempfehlungen bezüglich NS existieren nicht. Glukokortikoide werden analog zur pulmonalen Sarkoidose als First-line-Therapie eingesetzt. Je nach Schweregrad der Beschwerden wird zudem zuvor oft ein Kortisonstoß (z. B. 1 g Methylprednisolon pro Tag für 3 bis 5 Tage) verabreicht. Bei unzureichendem Therapieansprechen oder Rezidiv wird bevorzugt der zusätzliche Einsatz von Methotrexat empfohlen. Alternativen bei Therapieversagen sind Azathioprin, Hydroxychloroquin, Mycophenolat oder Infliximab [40].

Bei Vorliegen einer krankheitsassoziierten (Small-fibre‑)Polyneuropathie kann unter etablierter immunmodulatorischer/-suppressiver Therapie eine Stabilisierung oder Besserung eintreten. Zur Behandlung persistierender neuropathischer Schmerzen bestehen keine gesonderten Empfehlungen, es sollen analog zur Behandlung anderer neuropathischer Schmerzen Antikonvulsiva, Antidepressiva, Opiate und Lokalanästhetika angewandt werden [120].

## Gastrointestinale und hepatale Sarkoidose

Eine klinisch manifeste Beteiligung des Gastrointestinaltrakts bei Sarkoidose ist äußerst selten (< 1 %), wohingegen eine symptomatische Beteiligung der Leber mit 5–20 % häufiger zu beobachten ist. Die Rate an subklinischen Beteiligungen der Organe aus dem Gastrointestinaltrakt ist wesentlich höher.

### Gastrointestinale Sarkoidose

Eine gastrointestinale Beteiligung im Rahmen einer Sarkoidose kann vom Mund bis zum Rektum auftreten [121]. Der obere Gastrointestinaltrakt (insbesondere der Magen) scheint hier häufiger betroffen zu sein als der untere [122].

Im Mund kann sich die Sarkoidose als Knoten oder Ulzerationen im Bereich der bukkalen Schleimhaut oder des Zahnfleisches als Gingivahyperplasie bzw. Gingivitis manifestieren. Ein Mitbeteiligung der Ösophagusschleimhaut und/oder -muskulatur betrifft meist das untere Ösophagusdrittel und kann zur Dys- und/oder Odynophagie bis hin zu einem Achalasie-ähnlichen Bild führen [122]. Die endoskopisch sichtbaren Veränderungen sind meist unspezifisch (plaqueartige oder knotig) und bedürfen einer bioptischen Abklärung [123].

Die Magenbeteiligung ist durch Vorliegen einer granulomatösen Gastritis charakterisiert. Wichtige Differenzialdiagnosen sind der Morbus Crohn oder andere Formen der chronischen Gastritis, da diese ebenfalls zu Granulomen in der Magenschleimhaut führen können. Die Magenbeteiligung ist meist ein Zufallsbefund: Dyspeptische Beschwerden oder Komplikationen wie eine gastrointestinale Blutung oder Obstruktion sind selten [121, 122]. Patienten mit bekannter Sarkoidose, die entsprechende Symptome aufweisen, sollten endoskopisch abgeklärt werden [122]. Den dyspeptischen Symptomen kann – obwohl kontrollierte Studien hierzu fehlen – mit Protonenpumpenhemmern entgegengewirkt werden [121, 122, 124]. Eine Beteiligung von Dünndarm, Kolon, Rektum und Pankreas ist äußerst selten.

### Leberbeteiligung

Eine Leberbeteiligung bei Sarkoidose kommt häufig vor (50–90 %), ist aber nur in 5–15 % symptomatisch [125]. Das klinische Spektrum kann von einer asymptomatischen Erhöhung der Leberenzyme (vorrangig cholestatisches Muster mit Erhöhung der alkalischen Phosphatase und Gamma-Glutamyl-Transferase) bis hin zu Hepatomegalie, Schmerzen im rechten Oberbauch und Juckreiz reichen. Das morphologische Erscheinungsbild der hepatalen Sarkoidose kann dem einer primär sklerosierenden Cholangitis (intrahepatische biliäre Strikturen) oder einer primär biliären Cholangitis ähneln [126], aber auch intrahepatische Raumforderungen (meist in Nahbeziehung zu Pfortaderästen, in der Regel bis maximal 3 cm) können auftreten [127]. Zur Entwicklung einer biliären Fibrose oder Zirrhose mit portaler Hypertension kommt es nur selten (bei 6–8 %) [121]. Die Diagnosestellung erfolgt auch hier histologisch und erfordert den Ausschluss anderer Ursachen von Granulomen in der Leber (z. B. TBC, granulomatöse Hepatitis im Rahmen einer „drug-induced liver injury“, primär biliäre Cholangitis, andere Autoimmunerkrankungen wie Vaskulitiden …) [128]. Neben der Standardtherapie der Sarkoidose kann im Falle einer intrahepatischen Cholestase eine empirische Therapie mit Ursodeoxycholsäure 13–15(–20) mg/kg zur Verbesserung der Cholestaseparameter führen [129–131].

Eine Beteiligung der Milz bei Sarkoidose ist häufig und meist asymptomatisch. Bei bis zu 80 % der Patienten liegt eine Splenomegalie vor, auch Rundherde in der Milz können in der Bildgebung detektiert werden [110].

## Rehabilitation

Eine multimodale interprofessionelle Rehabilitation wird je nach Leitsymptom/-organ (Lunge/Herz/Neuro/Bewegungs- und Stützapparat) über die Sozialversicherungsträger ambulant bzw. stationär beantragt und hat nachgewiesene positive Effekte bei Patienten mit Sarkoidose. So kann die Leistungsfähigkeit, bemessen am 6‑Minuten-Gehtest, verbessert werden [132], ähnliche Daten gibt es auch zu Kraftzuwächsen der unteren Extremität. Das Leitsymptom Fatigue, welches mit eingeschränkter Lebensqualität, kognitiven Defiziten bis hin zu Depressionen einhergehen kann, konnte ebenfalls signifikant verbessert werden. Eine prospektive deutsche Multicenterstudie [133] und eine Studie zu Langzeiteffekten der Reha [134] konnten diese Daten bestätigen – hier wurden zusätzlich Daten zur Lebensqualität erhoben, die ebenso positiv waren. Für die Sozialversicherungsträger waren positive Aspekte für die Arbeitsfähigkeit darstellbar, was entscheidend ist, da Sarkoidosepatienten üblicherweise im erwerbsfähigen Alter erstdiagnostiziert werden. Auch eine Zuweisung zu einer Rehabilitation mit rheumatologischem Schwerpunkt sollte in entsprechend gearteten Fällen erwogen werden.

Aufgrund der Komplexität der Erkrankung ist bei Sarkoidose eine sehr genaue prä-/rehabilitative Diagnostik notwendig und hat entsprechende Konsequenzen: Ein Schlafapnoescreening kann indiziert sein, ebenso kann eine belastungsinduzierte Hypoxämie bzw. eine Diffusionsstörung demaskiert werden, welche therapeutisch zu einem Intervalltraining führen sollte [135]. Eine kardiale Mitbeteiligung bedingt eine Umstellung des Settings im Sinne einer rhythmologischen Überwachung, und eine pulmonale Hypertonie würde ebenso eine deutliche Änderung der medizinischen Trainingstherapie nach sich ziehen. Neurokognitive Veränderungen bedingen eine ergotherapeutische Intervention, darüber hinaus können eine psychologische Betreuung [136] und sozialmedizinische Maßnahmen notwendig sein.

Ebenso werden Schulungen im Sinne einer ausführlichen Erklärung der Erkrankung von Patienten als sehr hilfreich empfunden.

## Patientenperspektive in Bezug auf Symptome, Diagnose und Patientenpfade in der Abklärung

Je nach Krankheitsschwere und initialem Manifestationsort ergeben sich unterschiedliche Latenzzeiten von den ersten Symptomen bis hin zur Diagnose und daher variable Patientenpfade. Bei der akuten Sarkoidose oder der Hautsarkoidose ergibt sich der Erkrankungsverdacht oft schon durch Blickdiagnose. Vielfach präsentieren Betroffene aber unspezifische Symptome, und es vergehen oft mehrere Jahre mit Konsultation vieler Ärzte bis zur richtigen Diagnose. In diesen Fällen fehlt auch oft ein „Leitfaden“ durch das Gesundheitssystem, da für Sarkoidose hierzulande eine standardisierte, strukturierte Versorgungsstruktur wie auch bei anderen seltenen und chronischen Erkrankungen fehlt. Auch kommt es in diesem Stadium aufgrund der oft wechselnden und unspezifischen Beschwerden zu Fehldiagnosen (wie Asthma, Burn-out, Depression) [137].

Ist die Diagnose Sarkoidose etabliert, werden Patienten immer noch häufig mit „Mindermeinungen“ konfrontiert, z. B. dass Sarkoidose eine „gutartige“ Erkrankung sei, die meist von selber verschwinde, oder dass bei Sarkoidose immer die Lunge mitbetroffen sei und andere Beschwerden keinen relevanten Krankheitswert hätten. Besonders problematisch ist, dass Sarkoidose häufig in der Mitte des Lebens auftritt, zu einer Zeit in der soziales, familiäres und berufliches „Funktionieren“ besonders gefordert werden. In dieser Situation ist es umso belastender, krank oder leistungsunfähig zu sein, während z. B. Lungenfunktionstests oft normale Werte zeigen [13]. Die Anwesenheit von Granulomen in Organen erklärt bei Sarkoidose nicht alle Symptome: Es gibt neben organspezifischen Auffälligkeiten, die durch Funktionstests und Labor überprüft werden können, eine Vielzahl allgemeiner Symptome wie Fatigue, Fieber, Anorexie, Arthralgie, Muskelschmerzen, kognitives Versagen und Schwäche. Obwohl sie speziell aus Patientensicht wesentliche Bestandteile des Krankheitsbildes der Sarkoidose sind, werden diese Symptome oft nicht als solche erkannt, beachtet oder dokumentiert [34, 138]. Fatigue ist hierbei wahrscheinlich das am meisten einschränkende Problem des täglichen Lebens vieler Sarkoidosepatienten [139], die damit einhergehende Leistungslimitierung wird von außen oft unterschätzt [140]. Als Prioritäten von Patienten bei ihrer Behandlung gelten nicht Lungenfunktion oder Bildgebung, sondern Besserung/Erhalt ihrer Lebensqualität und Funktionieren im Alltag [32].

Bei einem komplexeren Verlauf, wenn mehrere Organe betroffen sind, ist es empfehlenswert, die Patienten an einem interdisziplinären Zentrum zu betreuen, an dem die verschiedenen Fachrichtungen unter einem Dach vereint sind und an dem Expertise für seltene Krankheiten besteht. Idealerweise haben diese Zentren auch Möglichkeiten, die Patienten in ihrem Erkrankungsbild zu schulen [31, 37, 141].

Ein interdisziplinäres Zentrum mit hoher Fallzahl ermöglicht das Erkennen und die anspruchsvolle Betreuung auch von seltenen Krankheitskonstellationen nach internationalen Standards [4]. Daneben braucht es aber natürlich auch eine gute Anbindung an Hausarzt und betreuende niedergelassene Fachärzte als Basis der Versorgung und idealerweise als „Vertrauensärzte“ neben dezidierten Spezialisten mit naturgemäß begrenzten Ressourcen.

## Fazit und Ausblick

Seit ihrer Erstbeschreibung im Jahr 1869 durch Jonathan Hutchinson [142] ist die Sarkoidose Gegenstand medizinischer Forschung, jedoch verbleiben weiterhin offene Fragen bezüglich exakter Pathogenese, optimaler Therapie und prognostisch unterschiedlichen Verläufen [4].

Genetische Varianten [4], mTOR Pathway [25], die Beteiligung von Zytokinen, T‑Helferzellen sowie Januskinasen [4, 56] sind nur einige Bereiche aktueller pathophysiologischer Forschung und Therapieentwicklung [4]. Die klinische Variabilität der Sarkoidose ist hoch und führt oft zu unklaren Verläufen und unsicheren Prognosen [4]. Eine Differenzierungsmöglichkeit bietet eine klinische Phänotypisierung nach führender Organbeteiligung (Auge-Haut-Herz-ZNS, muskuloskeletal-Haut, Lunge-Lymphknoten, gastrointestinal), was auch Implikationen für die Therapie hat [1, 143].

**Abb. 6:**
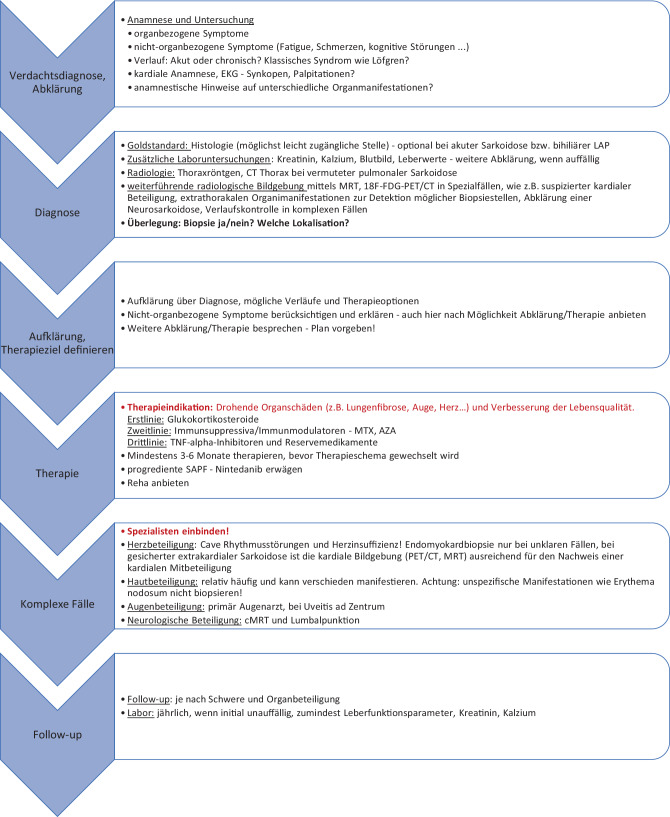
„Bullet points“ für Anamnese, Diagnose, Therapie und Verlauf der Sarkoidose

Die medikamentöse Behandlung, bestehend aus Steroiden und Immunsuppressiva, ist weithin akzeptiert und auch relativ standardisiert, jedoch häufig mit Nebenwirkungen verbunden [40]. Es bestehen internationale Leitlinien und Positionspapiere zur Therapie [13, 40, 107], die optimalen Dosierungen, die Therapiedauer und die Möglichkeit von Kombinationstherapien sind jedoch noch nicht ausreichend evidenzbasiert geklärt. Gerade bei einer sich so variabel manifestierenden und häufig chronisch verlaufenden Erkrankung, die mit einer hohen und oft polytopen Beschwerdesymptomatik einhergehen kann, sind Patientenschulung, Aufklärung und „shared decision-making“ sowie multidisziplinäre Betreuung nach standardisierten Abläufen unerlässlich. Sowohl aus Sicht der Patienten als auch der Behandler gibt es jedenfalls für die Zukunft einen hohen Bedarf an Forschung in der Klinik und an der Basis sowie auch an Optimierung der bestehenden Abläufe in der Beziehung zwischen Patienten, betreuenden extramuralen Ärzten und Spezialisten in Zentren. Dieses Positionspapier soll als Versuch verstanden werden, diesen Prozess der Vernetzung und Weiterentwicklung anzustoßen und ein Stück weiterzuführen (Abb. 6).

## References

[1] Schupp JC, Freitag-Wolf S, Bargagli E, et al. Phenotypes of organ involvement in sarcoidosis. Eur Respir J. 2018; 10.1183/13993003.00991-2017. 29371378 10.1183/13993003.00991-2017

[2] Ungprasert P, Carmona EM, Utz JP, Ryu JH, Crowson CS, Matteson EL. Epidemiology of sarcoidosis 1946–2013: a population-based study. Mayo Clin Proc. 2016;91(2):183–8. 10.1016/j.mayocp.2015.10.024. 26727158 10.1016/j.mayocp.2015.10.024 PMC4744129

[3] Grunewald J, Grutters JC, Arkema EV, Saketkoo LA, Moller DR, Müller-Quernheim J. Sarcoidosis. Nat Rev Dis Primers. 2019;5(1):45. 10.1038/s41572-019-0096-x. 31273209 10.1038/s41572-019-0096-x

[4] Drent M, Crouser ED, Grunewald J. Challenges of sarcoidosis and its management. N Engl J Med. 2021;385(11):1018–32. 10.1056/NEJMra2101555. 34496176 10.1056/NEJMra2101555

[5] Arkema EV, Cozier YC. Sarcoidosis epidemiology: recent estimates of incidence, prevalence and risk factors. Curr Opin Pulm Med. 2020;26(5):527–34. 10.1097/MCP.0000000000000715. 32701677 10.1097/MCP.0000000000000715 PMC7755458

[6] Prasse A. The diagnosis, differential diagnosis, and treatment of sarcoidosis. Dtsch Arztebl Int. 2016;113(33):565–74. 10.3238/arztebl.2016.0565. 27598883 10.3238/arztebl.2016.0565 PMC5015588

[7] Baughman RP, Field S, Costabel U, et al. Sarcoidosis in america. Analysis based on health care use. Ann Am Thorac Soc. 2016;13(8):1244–52. 10.1513/AnnalsATS.201511-760OC. 27509154 10.1513/AnnalsATS.201511-760OC

[8] Crouser ED, Maier LA, Wilson KC, et al. Diagnosis and detection of sarcoidosis. An official American thoracic society clinical practice guideline. Am J Respir Crit Care Med. 2020;201(8):e26–e51. 10.1164/rccm.202002-0251ST. 32293205 10.1164/rccm.202002-0251ST PMC7159433

[9] Pagán AJ, Ramakrishnan L. The formation and function of granulomas. Annu Rev Immunol. 2018;36:639–65. 10.1146/annurev-immunol-032712-100022. 29400999 10.1146/annurev-immunol-032712-100022

[10] Meyer KC, Raghu G, Baughman RP, et al. An official American thoracic society clinical practice guideline: the clinical utility of bronchoalveolar lavage cellular analysis in interstitial lung disease. Am J Respir Crit Care Med. 2012;185(9):1004–14. 10.1164/rccm.201202-0320ST. 22550210 10.1164/rccm.201202-0320ST

[11] Kraaijvanger R, Janssen Bonás M, Vorselaars ADM, Veltkamp M. Biomarkers in the diagnosis and prognosis of sarcoidosis: current use and future prospects. Front Immunol. 2020;11:1443. 10.3389/fimmu.2020.01443. 32760396 10.3389/fimmu.2020.01443 PMC7372102

[12] Valeyre D, Prasse A, Nunes H, Uzunhan Y, Brillet P‑Y, Müller-Quernheim J. Sarcoidosis. Lancet. 2014;383(9923):1155–67. 10.1016/S0140-6736(13)60680-7. 24090799 10.1016/S0140-6736(13)60680-7

[13] Thillai M, Atkins CP, Crawshaw A, et al. BTS clinical statement on pulmonary sarcoidosis. Thorax. 2021;76(1):4–20. 10.1136/thoraxjnl-2019-214348. 33268456 10.1136/thoraxjnl-2019-214348

[14] Baughman RP, Janovcik J, Ray M, Sweiss N, Lower EE. Calcium and vitamin D metabolism in sarcoidosis. Sarcoidosis. 2013;30(2):113–20. 24071882

[15] Scadding JG. Prognosis of intrathoracic sarcoidosis in England. A review of 136 cases after five years’ observation. Br Med J. 1961;2(5261):1165–72. 10.1136/bmj.2.5261.1165. 14497750 10.1136/bmj.2.5261.1165 PMC1970202

[16] Bandyopadhyay D, Mirsaeidi MS. Sarcoidosis-associated pulmonary fibrosis: joining the dots. Eur Respir Rev. 2023; 10.1183/16000617.0085-2023. 37758275 10.1183/16000617.0085-2023 PMC10523156

[17] Desai SR, Sivarasan N, Johannson KA, et al. High-resolution CT phenotypes in pulmonary sarcoidosis: a multinational Delphi consensus study. Lancet Respir Med. 2023; 10.1016/S2213-2600(23)00267-9. 38104579 10.1016/S2213-2600(23)00267-9

[18] Judson MA. The management of sarcoidosis in the 2020s by the primary care physician. Am J Med. 2023;136(6):534–44. 10.1016/j.amjmed.2023.02.014. 36889493 10.1016/j.amjmed.2023.02.014

[19] Keijsers RGM, Grutters JC. In which patients with sarcoidosis is FDG PET/CT indicated? J Clin Med. 2020; 10.3390/jcm9030890. 32213991 10.3390/jcm9030890 PMC7141490

[20] Treglia G, Annunziata S, Sobic-Saranovic D, Bertagna F, Caldarella C, Giovanella L. The role of 18F-FDG-PET and PET/CT in patients with sarcoidosis: an updated evidence-based review. Acad Radiol. 2014;21(5):675–84. 10.1016/j.acra.2014.01.008. 24703482 10.1016/j.acra.2014.01.008

[21] Blankstein R, Waller AH. Evaluation of known or suspected cardiac sarcoidosis. Circ Cardiovasc Imaging. 2016;9(3):e867. 10.1161/CIRCIMAGING.113.000867. 26926267 10.1161/CIRCIMAGING.113.000867

[22] Judson MA. Environmental risk factors for sarcoidosis. Front Immunol. 2020;11:1340. 10.3389/fimmu.2020.01340. 32676081 10.3389/fimmu.2020.01340 PMC7333358

[23] Rossides M, Grunewald J, Eklund A, et al. Familial aggregation and heritability of sarcoidosis: a Swedish nested case-control study. Eur Respir J. 2018; 10.1183/13993003.00385-2018. 29946010 10.1183/13993003.00385-2018

[24] Rybicki BA, Iannuzzi MC, Frederick MM, et al. Familial aggregation of sarcoidosis. A case-control etiologic study of sarcoidosis (ACCESS). Am J Respir Crit Care Med. 2001;164(11):2085–91. 10.1164/ajrccm.164.11.2106001. 11739139 10.1164/ajrccm.164.11.2106001

[25] Linke M, Pham HTT, Katholnig K, et al. Chronic signaling via the metabolic checkpoint kinase mTORC1 induces macrophage granuloma formation and marks sarcoidosis progression. Nat Immunol. 2017;18(3):293–302. 10.1038/ni.3655. 28092373 10.1038/ni.3655 PMC5321578

[26] Pacheco Y, Lim CX, Weichhart T, Valeyre D, Bentaher A, Calender A. Sarcoidosis and the mTOR, Rac1, and autophagy triad. Trends Immunol. 2020;41(4):286–99. 10.1016/j.it.2020.01.007. 32122794 10.1016/j.it.2020.01.007

[27] Pizzini A, Bacher H, Aichner M, et al. High expression of mTOR signaling in granulomatous lesions is not predictive for the clinical course of sarcoidosis. Respir Med. 2021;177:106294. 10.1016/j.rmed.2020.106294. 33485108 10.1016/j.rmed.2020.106294

[28] Bueno-Beti C, Lim CX, Protonotarios A, et al. An mTORC1-dependent mouse model for cardiac sarcoidosis. J Am Heart Assoc. 2023;12(19):e30478. 10.1161/JAHA.123.030478. 37750561 10.1161/JAHA.123.030478 PMC10727264

[29] Baughman RP, Judson MA, Wells A. The indications for the treatment of sarcoidosis: Wells Law. Sarcoidosis. 2017;34(4):280–2. 10.36141/svdld.v34i4.6957. 10.36141/svdld.v34i4.6957 PMC7170078 32476859

[30] Hu X, Carmona EM, Yi ES, Pellikka PA, Ryu J. Causes of death in patients with chronic sarcoidosis. Sarcoidosis. 2016;33(3):275–80. 27758994

[31] Moor CC, van Manen MJG, van Hagen PM, et al. Needs, perceptions and education in sarcoidosis: a live interactive survey of patients and partners. Lung. 2018;196(5):569–75. 10.1007/s00408-018-0144-4. 30088094 10.1007/s00408-018-0144-4 PMC6153596

[32] Baughman RP, Barriuso R, Beyer K, et al. Sarcoidosis: patient treatment priorities. ERJ Open Res. 2018; 10.1183/23120541.00141-2018. 30588477 10.1183/23120541.00141-2018 PMC6302206

[33] Patel AS, Siegert RJ, Creamer D, et al. The development and validation of the King’s sarcoidosis questionnaire for the assessment of health status. Thorax. 2013;68(1):57–65. 10.1136/thoraxjnl-2012-201962. 23065052 10.1136/thoraxjnl-2012-201962

[34] Drent M, Costabel U, Crouser ED, Grunewald J, Bonella F. Misconceptions regarding symptoms of sarcoidosis. Lancet Respir Med. 2021;9(8):816–8. 10.1016/S2213-2600(21)00311-8. 34216548 10.1016/S2213-2600(21)00311-8

[35] Bosse-Henck A, Wirtz H, Hinz A. Organ manifestation and subjective sleep quality in sarcoidosis. Pneumologie. 2016;70(8):522–9. 10.1055/s-0042-100554. 26894478 10.1055/s-0042-100554

[36] Hendriks C, Drent M, De Kleijn W, Elfferich M, Wijnen P, De Vries J. Everyday cognitive failure and depressive symptoms predict fatigue in sarcoidosis: a prospective follow-up study. Respir Med. 2018;138:S24–S30. 10.1016/j.rmed.2017.11.008. 10.1016/j.rmed.2017.11.008 29239767

[37] Moor CC, Kahlmann V, Culver DA, Wijsenbeek MS. Comprehensive care for patients with sarcoidosis. J Clin Med. 2020; 10.3390/jcm9020390. 32024123 10.3390/jcm9020390 PMC7074229

[38] Saketkoo LA, Russell A‑M, Jensen K, et al. Health-related quality of life (HRQoL) in sarcoidosis: diagnosis, management, and health outcomes. Diagnostics. 2021; 10.3390/diagnostics11061089. 34203584 10.3390/diagnostics11061089 PMC8232334

[39] Kirkil G, Lower EE, Baughman RP. Predictors of mortality in pulmonary sarcoidosis. Chest. 2018;153(1):105–13. 10.1016/j.chest.2017.07.008. 28728933 10.1016/j.chest.2017.07.008

[40] Baughman RP, Valeyre D, Korsten P, et al. ERS clinical practice guidelines on treatment of sarcoidosis. Eur Respir J. 2021; 10.1183/13993003.04079-2020. 34140301 10.1183/13993003.04079-2020

[41] Swigris JJ, Olson AL, Huie TJ, et al. Sarcoidosis-related mortality in the United States from 1988 to 2007. Am J Respir Crit Care Med. 2011;183(11):1524–30. 10.1164/rccm.201010-1679OC. 21330454 10.1164/rccm.201010-1679OC PMC3137141

[42] Trivieri MG, Spagnolo P, Birnie D, et al. Challenges in cardiac and pulmonary sarcoidosis. J Am Coll Cardiol. 2020;76(16):1878–901. 10.1016/j.jacc.2020.08.042. 33059834 10.1016/j.jacc.2020.08.042 PMC7808240

[43] Belperio JA, Shaikh F, Abtin FG, et al. Diagnosis and treatment of pulmonary sarcoidosis: a review. JAMA. 2022;327(9):856–67. 35230389 10.1001/jama.2022.1570

[44] Spagnolo P, Rossi G, Trisolini R, Sverzellati N, Baughman RP, Wells AU. Pulmonary sarcoidosis. Lancet Respir Med. 2018;6(5):389–402. 10.1016/S2213-2600(18)30064-X. 29625772 10.1016/S2213-2600(18)30064-X

[45] Huitema MP, Mathijssen H, Mager JJ, Snijder RJ, Grutters JC, Post MC. Sarcoidosis-associated pulmonary hypertension. Semin Respir Crit Care Med. 2020;41(5):659–72. 10.1055/s-0040-1713615. 32777851 10.1055/s-0040-1713615

[46] Khan NA, Donatelli CV, Tonelli AR, et al. Toxicity risk from glucocorticoids in sarcoidosis patients. Respir Med. 2017;132:9–14. 10.1016/j.rmed.2017.09.003. 29229111 10.1016/j.rmed.2017.09.003

[47] Dhooria S, Sehgal IS, Agarwal R, et al. High-dose (40 mg) versus low-dose (20 mg) prednisolone for treating sarcoidosis: a randomised trial (SARCORT trial). Eur Respir J. 2023; 10.1183/13993003.00198-2023. 37690784 10.1183/13993003.00198-2023

[48] Rahaghi FF, Baughman RP, Saketkoo LA, et al. Delphi consensus recommendations for a treatment algorithm in pulmonary sarcoidosis. Eur Respir Rev. 2020; 10.1183/16000617.0146-2019. 32198218 10.1183/16000617.0146-2019 PMC9488897

[49] Humphrey MB, Russell L, Danila MI, et al. 2022 American college of rheumatology guideline for the prevention and treatment of glucocorticoid-induced osteoporosis. Arthritis Rheumatol. 2023;75(12):2088–102. 10.1002/art.42646. 37845798 10.1002/art.42646

[50] Baughman RP, Cremers JP, Harmon M, Lower EE, Drent M. Methotrexate in sarcoidosis: hematologic and hepatic toxicity encountered in a large cohort over a six year period. Sarcoidosis. 2020;37(3):e2020001. 10.36141/svdld.v37i3.9362. 10.36141/svdld.v37i3.9362 PMC7690061 33264378

[51] Pande A, Culver DA. Knowing when to use steroids, immunosuppressants or biologics for the treatment of sarcoidosis. Expert Rev Respir Med. 2020;14(3):285–98. 10.1080/17476348.2020.1707672. 31868547 10.1080/17476348.2020.1707672

[52] Baughman RP, Drent M, Kavuru M, et al. Infliximab therapy in patients with chronic sarcoidosis and pulmonary involvement. Am J Respir Crit Care Med. 2006;174(7):795–802. 10.1164/rccm.200603-402OC. 16840744 10.1164/rccm.200603-402OC

[53] Sweiss NJ, Noth I, Mirsaeidi M, et al. Efficacy results of a 52-week trial of adalimumab in the treatment of refractory sarcoidosis. Sarcoidosis. 2014;31(1):46–54. PMC4134103 24751453

[54] Flaherty KR, Wells AU, Cottin V, et al. Nintedanib in progressive fibrosing interstitial lung diseases. N Engl J Med. 2019;381(18):1718–27. 10.1056/NEJMoa1908681. 31566307 10.1056/NEJMoa1908681

[55] Raghu G, Remy-Jardin M, Richeldi L, et al. Idiopathic pulmonary fibrosis (an update) and progressive pulmonary fibrosis in adults: an official ATS/ERS/JRS/ALAT clinical practice guideline. Am J Respir Crit Care Med. 2022;205(9):e18–e47. 10.1164/rccm.202202-0399ST. 35486072 10.1164/rccm.202202-0399ST PMC9851481

[56] Damsky W, Wang A, Kim DJ, et al. Inhibition of type 1 immunity with tofacitinib is associated with marked improvement in longstanding sarcoidosis. Nat Commun. 2022;13(1):3140. 10.1038/s41467-022-30615-x. 35668129 10.1038/s41467-022-30615-x PMC9170782

[57] Wang A, Rahman N‑T, McGeary MK, et al. Treatment of granuloma annulare and suppression of proinflammatory cytokine activity with tofacitinib. J Allergy Clin Immunol. 2021;147(5):1795–809. 10.1016/j.jaci.2020.10.012. 33317858 10.1016/j.jaci.2020.10.012

[58] Damsky W, Young BD, Sloan B, Miller EJ, Obando JA, King B. Treatment of multiorgan sarcoidosis with tofacitinib. ACR Open Rheumatol. 2020;2(2):106–9. 10.1002/acr2.11112. 31916703 10.1002/acr2.11112 PMC7011417

[59] Liu J, Ma P, Lai L, et al. Transcriptional and immune landscape of cardiac sarcoidosis. Circ Res. 2022;131(8):654–69. 10.1161/CIRCRESAHA.121.320449. 36111531 10.1161/CIRCRESAHA.121.320449 PMC9514756

[60] Lim CX, Redl A, Kleissl L, et al. Aberrant lipid metabolism in macrophages is associated with granuloma formation in sarcoidosis. Am J Respir Crit Care Med. 2024; 10.1164/rccm.202307-1273OC. 38353578 10.1164/rccm.202307-1273OC PMC7617514

[61] Redl A, Doberer K, Unterluggauer L, et al. Efficacy and safety of mTOR inhibition in cutaneous sarcoidosis: a single-centre trial. Lancet Rheumatol. 2024;6(2):e81–e91. 10.1016/S2665-9913(23)00302-8.38267106 10.1016/S2665-9913(23)00302-8

[62] Gupta N, Bleesing JH, McCormack FX. Successful response to treatment with sirolimus in pulmonary sarcoidosis. Am J Respir Crit Care Med. 2020;202(9):e119–e20. 10.1164/rccm.202004-0914IM. 32730705 10.1164/rccm.202004-0914IM

[63] Lee GM, Pope K, Meek L, Chung JH, Hobbs SB, Walker CM. Sarcoidosis: a diagnosis of exclusion. AJR Am J Roentgenol. 2020;214(1):50–8. 10.2214/AJR.19.21436. 31670585 10.2214/AJR.19.21436

[64] Kollert F, Geck B, Suchy R, et al. The impact of gas exchange measurement during exercise in pulmonary sarcoidosis. Respir Med. 2011;105(1):122–9. 10.1016/j.rmed.2010.09.007. 20926272 10.1016/j.rmed.2010.09.007

[65] Esteves TC, Aparicio G, Ferrer B, Garcia-Patos V. Prognostic value of skin lesions in sarcoidosis: clinical and histopathological clues. Eur J Dermatol. 2015;25(6):556–62. 10.1684/ejd.2015.2666. 26553412 10.1684/ejd.2015.2666

[66] Caplan A, Rosenbach M, Imadojemu S. Cutaneous sarcoidosis. Semin Respir Crit Care Med. 2020;41(5):689–99. 10.1055/s-0040-1713130. 32593176 10.1055/s-0040-1713130

[67] Haimovic A, Sanchez M, Judson MA, Prystowsky S. Sarcoidosis: a comprehensive review and update for the dermatologist: part I. Cutaneous disease. J Am Acad Dermatol. 2012;66(5):699.e1–699.18. 10.1016/j.jaad.2011.11.965. quiz 717–718. 22507585 10.1016/j.jaad.2011.11.965

[68] Giner T, Benoit S, Kneitz H, Goebeler M. Sarcoidosis : dermatological view of a rare multisystem disease. Hautarzt. 2017;68(7):526–35. 10.1007/s00105-017-4005-5. 28573316 10.1007/s00105-017-4005-5

[69] Sweiss NJ, Patterson K, Sawaqed R, et al. Rheumatologic manifestations of sarcoidosis. Semin Respir Crit Care Med. 2010;31(4):463–73. 10.1055/s-0030-1262214. 20665396 10.1055/s-0030-1262214 PMC3314339

[70] Bechman K, Christidis D, Walsh S, Birring SS, Galloway J. A review of the musculoskeletal manifestations of sarcoidosis. Rheumatology. 2018;57(5):777–83. 10.1093/rheumatology/kex317. 28968840 10.1093/rheumatology/kex317

[71] Kobak S. Sarcoidosis: a rheumatologist’s perspective. Ther Adv Musculoskelet Dis. 2015;7(5):196–205. 10.1177/1759720X15591310. 26425148 10.1177/1759720X15591310 PMC4572362

[72] Hammam N, Evans M, Morgan E, et al. Treatment of sarcoidosis in US rheumatology practices: data from the American college of rheumatology’s rheumatology Informatics system for effectiveness (RISE) registry. Arthritis Care Res. 2022;74(3):371–6. 10.1002/acr.24496. 10.1002/acr.24496 PMC8592780 33105057

[73] Lofgren S. Primary pulmonary sarcoidosis. I. Early signs and symptoms. Acta Med Scand. 1953;145(6):424–31. 13079656

[74] Visser H, Vos K, Zanelli E, et al. Sarcoid arthritis: clinical characteristics, diagnostic aspects, and risk factors. Ann Rheum Dis. 2002;61(6):499–504. 10.1136/ard.61.6.499. 12006321 10.1136/ard.61.6.499 PMC1754119

[75] Glennås A, Kvien TK, Melby K, et al. Acute sarcoid arthritis: occurrence, seasonal onset, clinical features and outcome. Br J Rheumatol. 1995;34(1):45–50. 10.1093/rheumatology/34.1.45. 7881838 10.1093/rheumatology/34.1.45

[76] Mañá J, Gómez-Vaquero C, Montero A, et al. Löfgren’s syndrome revisited: a study of 186 patients. Am J Med. 1999;107(3):240–5. 10.1016/s0002-9343(99)00223-5. 10492317 10.1016/s0002-9343(99)00223-5

[77] Abril A, Cohen MD. Rheumatologic manifestations of sarcoidosis. Curr Opin Rheumatol. 2004;16(1):51–5. 10.1097/00002281-200401000-00010. 14673389 10.1097/00002281-200401000-00010

[78] Boyaci B, Hornicek F, Rosenthal D, et al. Sarcoidosis of the spine: a report of five cases and a review of the literature. J Bone Joint Surg Am. 2012;94(7):e42. 10.2106/JBJS.K.00062. 22488626 10.2106/JBJS.K.00062

[79] Kobak S, Sever F, Ince O, Orman M. The prevalence of sacroiliitis and spondyloarthritis in patients with sarcoidosis. Int J Rheumatol. 2014;2014:289454. 10.1155/2014/289454. 24899899 10.1155/2014/289454 PMC4037117

[80] Awada H, Abi-Karam G, Fayad F. Musculoskeletal and other extrapulmonary disorders in sarcoidosis. Best Pract Res Clin Rheumatol. 2003;17(6):971–87. 10.1016/j.berh.2003.09.005. 15123046 10.1016/j.berh.2003.09.005

[81] Pitt P, Hamilton EB, Innes EH, Morley KD, Monk BE, Hughes GR. Sarcoid dactylitis. Ann Rheum Dis. 1983;42(6):634–9. 10.1136/ard.42.6.634. 6606399 10.1136/ard.42.6.634 PMC1001318

[82] Aptel S, Lecocq-Teixeira S, Olivier P, Regent D, Teixeira PG, Blum A. Multimodality evaluation of musculoskeletal sarcoidosis: Imaging findings and literature review. Diagn Interv Imaging. 2016;97(1):5–18. 10.1016/j.diii.2014.11.038. 25883076 10.1016/j.diii.2014.11.038

[83] Silverstein A, Siltzbach LE. Muscle involvement in sarcoidosis. Asymptomatic, myositis, and myopathy. Arch Neurol. 1969;21(3):235–41. 10.1001/archneur.1969.00480150025002. 5802453 10.1001/archneur.1969.00480150025002

[84] Ten Dam L, Raaphorst J, van der Kooi AJ, et al. Clinical characteristics and outcome in muscular sarcoidosis: a retrospective cohort study and literature review. Neuromuscul Disord. 2022;32(7):557–63. 10.1016/j.nmd.2022.05.012. 35654706 10.1016/j.nmd.2022.05.012

[85] Marcellis RGJ, Lenssen AF, Elfferich MDP, et al. Exercise capacity, muscle strength and fatigue in sarcoidosis. Eur Respir J. 2011;38(3):628–34. 21436356 10.1183/09031936.00117710

[86] Rosenbaum JT, Pasadhika S. Ocular sarcoidosis. Clin Chest Med. 2024;45(1):59–70. 10.1016/j.ccm.2023.08.003. 38245371 10.1016/j.ccm.2023.08.003

[87] Giorgiutti S, Jacquot R, El Jammal T, et al. Sarcoidosis-related uveitis: a review. J Clin Med. 2023; 10.3390/jcm12093194. 37176633 10.3390/jcm12093194 PMC10178951

[88] Mochizuki M, Smith JR, Takase H, Kaburaki T, Acharya NR, Rao NA. Revised criteria of international workshop on ocular sarcoidosis (IWOS) for the diagnosis of ocular sarcoidosis. Br J Ophthalmol. 2019;103(10):1418–22. 10.1136/bjophthalmol-2018-313356. 30798264 10.1136/bjophthalmol-2018-313356

[89] Dick AD, Rosenbaum JT, Al-Dhibi HA, et al. Guidance on noncorticosteroid systemic immunomodulatory therapy in noninfectious uveitis: fundamentals of care for UveitiS (FOCUS) initiative. Ophthalmology. 2018;125(5):757–73. 10.1016/j.ophtha.2017.11.017. 29310963 10.1016/j.ophtha.2017.11.017

[90] Humbert M, Kovacs G, Hoeper MM, et al. 2022 ESC/ERS guidelines for the diagnosis and treatment of pulmonary hypertension. Eur Heart J. 2022;43(38):3618–731. 10.1093/eurheartj/ehac237. 36017548 10.1093/eurheartj/ehac237

[91] Humbert M, Kovacs G, Hoeper MM, et al. 2022 ESC/ERS guidelines for the diagnosis and treatment of pulmonary hypertension. Eur Respir J. 2023; 10.1183/13993003.00879-2022. 37918877 10.1183/13993003.01726-2023

[92] Savale L, Huitema M, Shlobin O, et al. WASOG statement on the diagnosis and management of sarcoidosis-associated pulmonary hypertension. Eur Respir Rev. 2022; 10.1183/16000617.0165-2021. 35140103 10.1183/16000617.0165-2021 PMC9489049

[93] Boucly A, Cottin V, Nunes H, et al. Management and long-term outcomes of sarcoidosis-associated pulmonary hypertension. Eur Respir J. 2017; 10.1183/13993003.00465-2017. 29051269 10.1183/13993003.00465-2017

[94] Bandyopadhyay D, Humbert M. An update on sarcoidosis-associated pulmonary hypertension. Curr Opin Pulm Med. 2020;26(5):582–90. 10.1097/MCP.0000000000000701. 32740377 10.1097/MCP.0000000000000701

[95] Baughman RP, Culver DA, Cordova FC, et al. Bosentan for sarcoidosis-associated pulmonary hypertension: a double-blind placebo controlled randomized trial. Chest. 2014;145(4):810–7. 10.1378/chest.13-1766. 24177203 10.1378/chest.13-1766

[96] Baughman RP, Shlobin OA, Gupta R, et al. Riociguat for sarcoidosis-associated pulmonary hypertension: results of a 1-year double-blind, placebo-controlled trial. Chest. 2022;161(2):448–57. 10.1016/j.chest.2021.07.2162. 34363816 10.1016/j.chest.2021.07.2162 PMC9005858

[97] Le Pavec J, Valeyre D, Gazengel P, et al. Lung transplantation for sarcoidosis: outcome and prognostic factors. Eur Respir J. 2021; 10.1183/13993003.03358-2020. 33479107 10.1183/13993003.03358-2020

[98] . A joint procedural position statement on imaging in cardiac sarcoidosis: from the cardiovascular and inflammation & infection committees of the European association of nuclear medicine, the European association of cardiovascular imaging, and the American. Eur Heart J Cardiovasc Imaging. 2017;18(10):1073–89. 10.1093/ehjci/jex146. 10.1093/ehjci/jex146 28984894

[99] Birnie DH, Nery PB, Ha AC, Beanlands RSB. Cardiac sarcoidosis. J Am Coll Cardiol. 2016;68(4):411–21. 10.1016/j.jacc.2016.03.605. 27443438 10.1016/j.jacc.2016.03.605

[100] Seferović PM, Tsutsui H, McNamara DM, et al. Heart failure association of the ESC, heart failure society of america and Japanese heart failure society position statement on endomyocardial biopsy. Eur J Heart Fail. 2021;23(6):854–71. 10.1002/ejhf.2190. 34010472 10.1002/ejhf.2190

[101] Liang JJ, Hebl VB, DeSimone CV, et al. Electrogram guidance: a method to increase the precision and diagnostic yield of endomyocardial biopsy for suspected cardiac sarcoidosis and myocarditis. JACC Heart Fail. 2014;2(5):466–73. 10.1016/j.jchf.2014.03.015. 25194292 10.1016/j.jchf.2014.03.015 PMC4459501

[102] Vaidya VR, Abudan AA, Vasudevan K, et al. The efficacy and safety of electroanatomic mapping-guided endomyocardial biopsy: a systematic review. J Interv Card Electrophysiol. 2018;53(1):63–71. 10.1007/s10840-018-0410-7. 30003460 10.1007/s10840-018-0410-7

[103] Arbelo E, Protonotarios A, Gimeno JR, et al. 2023 ESC guidelines for the management of cardiomyopathies. Eur Heart J. 2023;44(37):3503–626. 10.1093/eurheartj/ehad194. 37622657 10.1093/eurheartj/ehad194

[104] Birnie DH, Sauer WH, Bogun F, et al. HRS expert consensus statement on the diagnosis and management of arrhythmias associated with cardiac sarcoidosis. Heart Rhythm. 2014;11(7):1305–23. 10.1016/j.hrthm.2014.03.043. 24819193 10.1016/j.hrthm.2014.03.043

[105] Slart RHJA, Glaudemans AWJM, Gheysens O, et al. Procedural recommendations of cardiac PET/CT imaging: standardization in inflammatory-, infective-, infiltrative-, and innervation- (4Is) related cardiovascular diseases: a joint collaboration of the EACVI and the EANM: summary. Eur Heart J Cardiovasc Imaging. 2020;21(12):1320–30. 10.1093/ehjci/jeaa299. 33245759 10.1093/ehjci/jeaa299 PMC7695243

[106] Hamzeh NY, Wamboldt FS, Weinberger HD. Management of cardiac sarcoidosis in the United States: a Delphi study. Chest. 2012;141(1):154–62. 10.1378/chest.11-0263. 21737493 10.1378/chest.11-0263 PMC3416033

[107] Skowasch D, Bonella F, Buschulte K, et al. Therapeutic pathways in sarcoidosisA position paper of the German society of respiratory medicine (DGP). Pneumologie. 2024;78(3):151–66. 10.1055/a-2259-1046. 38408486 10.1055/a-2259-1046

[108] Bergner R, Weiner SM, Kehl G, et al. Renal disease in sarcoidosis patients in a German multicentric retrospective cohort study. Respir Med. 2023;209:107121. 10.1016/j.rmed.2023.107121. 36669705 10.1016/j.rmed.2023.107121

[109] Gorsane I, Zammouri A, Hajji M, et al. Renal involvement in sarcoidosis: prognostic and predictive factors. Nephrol Ther. 2022;18(1):52–8. 10.1016/j.nephro.2021.08.001. 34756825 10.1016/j.nephro.2021.08.001

[110] Warshauer DM, Lee JKT. Imaging manifestations of abdominal sarcoidosis. AJR Am J Roentgenol. 2004;182(1):15–28. 10.2214/ajr.182.1.1820015. 14684507 10.2214/ajr.182.1.1820015

[111] Bergner R, Löffler C. Renal sarcoidosis: approach to diagnosis and management. Curr Opin Pulm Med. 2018;24(5):513–20. 10.1097/MCP.0000000000000504. 29965860 10.1097/MCP.0000000000000504

[112] Mehta S, Lightle A, Judson MA. Renal sarcoidosis. Nephrol Dial Transplant. 2023;38(4):803–10. 10.1093/ndt/gfac219. 35867874 10.1093/ndt/gfac219

[113] Mahévas M, Lescure FX, Boffa J‑J, et al. Renal sarcoidosis: clinical, laboratory, and histologic presentation and outcome in 47 patients. Medicine. 2009;88(2):98–106. 10.1097/MD.0b013e31819de50f. 19282700 10.1097/MD.0b013e31819de50f

[114] Hilderson I, Van Laecke S, Wauters A, Donck J. Treatment of renal sarcoidosis: is there a guideline? Overview of the different treatment options. Nephrol Dial Transplant. 2014;29(10):1841–7. 10.1093/ndt/gft442. 24235078 10.1093/ndt/gft442

[115] Fritz D, van de Beek D, Brouwer MC. Clinical features, treatment and outcome in neurosarcoidosis: systematic review and meta-analysis. BMC Neurol. 2016;16(1):220. 10.1186/s12883-016-0741-x. 27846819 10.1186/s12883-016-0741-x PMC5109654

[116] Bradshaw MJ, Pawate S, Koth LL, Cho TA, Gelfand JM. Neurosarcoidosis: pathophysiology, diagnosis, and treatment. Neurol Neuroimmunol Neuroinflamm. 2021; 10.1212/NXI.0000000000001084. 34607912 10.1212/NXI.0000000000001084 PMC8495503

[117] Pawate S, Moses H, Sriram S. Presentations and outcomes of neurosarcoidosis: a study of 54 cases. QJM. 2009;102(7):449–60. 10.1093/qjmed/hcp042. 19383611 10.1093/qjmed/hcp042

[118] Lexa FJ, Grossman RI. MR of sarcoidosis in the head and spine: spectrum of manifestations and radiographic response to steroid therapy. AJNR Am J Neuroradiol. 1994;15(5):973–82. 8059671 PMC8332168

[119] Vlad B, Neidhart S, Hilty M, Ziegler M, Jelcic I. Differentiating neurosarcoidosis from multiple sclerosis using combined analysis of basic CSF parameters and MRZ reaction. Front Neurol. 2023;14:1135392. 10.3389/fneur.2023.1135392. 37034091 10.3389/fneur.2023.1135392 PMC10080049

[120] Schlereth T. Guideline “diagnosis and non interventional therapy of neuropathic pain” of the German Society of Neurology (deutsche Gesellschaft für Neurologie). Neurol Res Pract. 2020;2:16. 10.1186/s42466-020-00063-3. 33324922 10.1186/s42466-020-00063-3 PMC7650069

[121] Shah N, Mitra A. Gastrointestinal and hepatic sarcoidosis: a review article. Clin Liver Dis. 2021;17(4):301–7. 10.1002/cld.1055. 10.1002/cld.1055 PMC8087901 33968393

[122] Brito-Zerón P, Bari K, Baughman RP, Ramos-Casals M. Sarcoidosis involving the gastrointestinal tract: diagnostic and therapeutic management. Am J Gastroenterol. 2019;114(8):1238–47. 10.14309/ajg.0000000000000171. 30865014 10.14309/ajg.0000000000000171

[123] Vahid B, Spodik M, Braun KN, Ghazi LJ, Esmaili A. Sarcoidosis of gastrointestinal tract: a rare disease. Dig Dis Sci. 2007;52(12):3316–20. 10.1007/s10620-006-9448-y. 17410465 10.1007/s10620-006-9448-y

[124] Ghrenassia E, Mekinian A, Chapelon-Albric C, et al. Digestive-tract sarcoidosis: French nationwide case-control study of 25 cases. Medicine. 2016;95(29):e4279. 10.1097/MD.0000000000004279. 27442665 10.1097/MD.0000000000004279 PMC5265782

[125] Judson MA. Extrapulmonary sarcoidosis. Semin Respir Crit Care Med. 2007;28(1):83–101. 10.1055/s-2007-970335. 17330194 10.1055/s-2007-970335

[126] . EASL clinical practice guidelines: management of cholestatic liver diseases. j Hepatol. 2009;51(2):237–67. 10.1016/j.jhep.2009.04.009. 10.1016/j.jhep.2009.04.009 19501929

[127] Scott GC, Berman JM, Higgins JLJ. CT patterns of nodular hepatic and splenic sarcoidosis: a review of the literature. J Comput Assist Tomogr. 1997;21(3):369–72. 10.1097/00004728-199705000-00006. 9135642 10.1097/00004728-199705000-00006

[128] Choi E‑YK, Lamps LW. Granulomas in the liver, with a focus on infectious causes. Surg Pathol Clin. 2018;11(2):231–50. 10.1016/j.path.2018.02.008. 29751872 10.1016/j.path.2018.02.008

[129] Alenezi B, Lamoureux E, Alpert L, Szilagyi A. Effect of ursodeoxycholic acid on granulomatous liver disease due to sarcoidosis. Dig Dis Sci. 2005;50(1):196–200. 10.1007/s10620-005-1300-2. 15712660 10.1007/s10620-005-1300-2

[130] Bakker GJ, Haan YCL, Maillette de Buy Wenniger LJ, Beuers U. Sarcoidosis of the liver: to treat or not to treat? Neth J Med. 2012;70(8):349–56. 23065982

[131] Cremers JP, Drent M, Baughman RP, Wijnen PA, Koek GH. Therapeutic approach of hepatic sarcoidosis. Curr Opin Pulm Med. 2012;18(5):472–82. 10.1097/MCP.0b013e3283541626. 22617809 10.1097/MCP.0b013e3283541626

[132] Strookappe B, Saketkoo LA, Elfferich M, et al. Physical activity and training in sarcoidosis: review and experience-based recommendations. Expert Rev Respir Med. 2016;10(10):1057–68. 10.1080/17476348.2016.1227244. 27552344 10.1080/17476348.2016.1227244

[133] Lingner H, Buhr-Schinner H, Hummel S, et al. Short-term effects of a multimodal 3‑week inpatient pulmonary rehabilitation programme for patients with sarcoidosis: the ProKaSaRe study. Respiration. 2018;95(5):343–53. 10.1159/000486964. 29486478 10.1159/000486964

[134] Wallaert B, Kyheng M, Labreuche J, Stelianides S, Wemeau L, Grosbois JM. Long-term effects of pulmonary rehabilitation on daily life physical activity of patients with stage IV sarcoidosis: a randomized controlled trial. Respir Med Res. 2020;77:1–7. 10.1016/j.resmer.2019.10.003. 31855785 10.1016/j.resmer.2019.10.003

[135] Gloeckl R, Zwick RH, Fürlinger U, et al. Prescribing and adjusting exercise training in chronic respiratory diseases—expert-based practical recommendations. Pulmonology. 2023;29(4):306–14. 10.1016/j.pulmoe.2022.09.004. 36272962 10.1016/j.pulmoe.2022.09.004

[136] Luu B, Gupta A, Fabiano N, et al. Influence of pulmonary rehabilitation on symptoms of anxiety and depression in interstitial lung disease: a systematic review of randomized controlled trials. Respir Med. 2023;219:107433. 10.1016/j.rmed.2023.107433. 37863339 10.1016/j.rmed.2023.107433

[137] European Association of Patients Organizations of Sarcoidosis and other Granulomatous Disorders (EPOS). EURORDIS sarcoidosis patient journey. 2021. https://ern-lung.eu/wp-content/uploads/2021/01/Sarc-PJ-Final.jpg..

[138] Drent M, Strookappe B, Hoitsma E, De Vries J. Consequences of sarcoidosis. Clin Chest Med. 2015;36(4):727–37. 10.1016/j.ccm.2015.08.013. 26593145 10.1016/j.ccm.2015.08.013

[139] Voortman M, Hendriks CMR, Elfferich MDP, et al. The burden of sarcoidosis symptoms from a patient perspective. Lung. 2019;197(2):155–61. 30778661 10.1007/s00408-019-00206-7 PMC6486948

[140] Hendriks CMR, Saketkoo LA, Elfferich MDP, De Vries J, Wijnen PAHM, Drent M. Sarcoidosis and work participation: the need to develop a disease-specific core set for assessment of work ability. Lung. 2019;197(4):407–13. 10.1007/s00408-019-00234-3. 31101981 10.1007/s00408-019-00234-3 PMC6647075

[141] European Lung Foundation. Sarcoidosis patient priorities. https://europeanlunginfo.org/sarcoidosis/library/..

[142] Hutchinson J. Anomalous diseases of skin and fingers. Illus Clin Surg. 1877; 10.2307/j.ctt9qh7zv.35.

[143] Baughman RP, Scholand MB, Rahaghi FF. Clinical phenotyping: role in treatment decisions in sarcoidosis. Eur Respir Rev. 2020; 10.1183/16000617.0145-2019. 32198217 10.1183/16000617.0145-2019 PMC9488542

