# Correction: Positionspapier der ÖGR und ÖGP zur Diagnose und Therapie der Sarkoidose 2024

**DOI:** 10.1007/s00508-024-02487-2

**Published:** 2024-12-16

**Authors:** Georg Sterniste, Klaus Hackner, Florentine Moazedi-Fürst, Marie Grasl, Marco Idzko, Guangyu Shao, Claudia Guttmann-Ducke, Emina Talakić, Helmut Prosch, Sylvia Lohfink-Schumm, Michael Gabriel, Clarice Lim, Johann Hochreiter, Brigitte Bucher, Barbara C. Böckle, Hans Peter Kiener, Christina Duftner, Kastriot Kastrati, Eva Rath, Marion Funk, Judith Löffler-Ragg, Monika Steinmaurer, Gabor Kovacs, Nicolas Verheyen, Holger Flick, Marlies Antlanger, Gerhard Traxler, Elisabeth Tatscher, Ralf Harun Zwick, David Lang

**Affiliations:** 1 Abteilung für Innere Medizin und Pneumologie, Klinik Floridsdorf, 1210 Wien, Österreich; 2 Klinische Abteilung für Pneumologie, Universitätsklinikum Krems, Karl Landsteiner Privatuniversität für Gesundheitswissenschaften, 3500 Krems, Österreich; 3 Klinische Abteilung für Rheumatologie und Immunologie, Medizinische Universität Graz, 8036 Graz, Österreich; 4 Abteilung für Atemwegs- und Lungenkrankheiten, Klinik Penzing, Ludwig Boltzmann Institut für Lungengesundheit, Wien, Österreich, 1140 Wien, Österreich; 5 Univ. Klinik für Innere Medizin II, Klin. Abteilung für Pulmologie, Medizinische Universität Wien, Wien, Österreich; 6 Klinische Abteilung für Allgemeine Radiologische Diagnostik, Universitätsklinik für Radiologie, Medizinische Universität Graz, Graz, Österreich; 7 Universitätsklinikum für Innere Medizin 4/Pneumologie, Kepler Universitätsklinikum, Johannes Kepler Universität, Linz, Österreich; 8 Univ. Klinik für Radiologie und Nuklearmedizin, Medizinische Universität Wien, Wien, Österreich; 9 Institut für Pathologie und Molekularpathologie, Kepler Universitätsklinikum, Johannes Kepler Universität, Linz, Österreich; 10 Institut für Nuklearmedizin und Endokrinologie, Kepler Universitätsklinikum, Johannes Kepler Universität, Linz, Österreich; 11 Lungenfibrose Forum Austria, Innermanzing, Österreich; 12 Abteilung Pneumologie, LKH Hochzirl Natters, Natters, Medizinische Universität Innsbruck, Innsbruck, Österreich; 13 Universitätsklinik für Dermatologie, Venerologie & Allergologie, Medizinische Universität Innsbruck, Innsbruck, Österreich; 14 Universitätsklinik für Innere Medizin III, Klinische Abteilung für Rheumatologie, Medizinische Universität Wien, Wien, Österreich; 15 Universitätsklinik für Innere Medizin II, Medizinische Universität Innsbruck, Innsbruck, Österreich; 16 1. Medizinische Abteilung, Hanusch Krankenhaus, Heinrich-Collin-Str. 30, 1140 Wien, Österreich; 17 Universitätsklinik für Augenheilkunde und Optometrie, Medizinische Universität Wien, Wien, Österreich; 18 Abteilung für Lungenkrankheiten, Klinikum Wels-Grieskirchen, 4600 Wels, Österreich; 19 Universitätsklinik für Innere Medizin, Klinische Abteilung für Pulmonologie, Medizinische Universität Graz, Graz, Österreich; 20 Universitätsklinik für Innere Medizin, Klinische Abteilung für Kardiologie, Medizinische Universität Graz, Graz, Österreich; 21 Universitätsklinik für Innere Medizin 2, Kepler Universitätsklinikum, Johannes Kepler Universität, Linz, Österreich; 22 Universitätsklinik für Neurologie, Kepler Universitätsklinikum, Johannes Kepler Universität, Linz, Österreich; 23 Universitätsklinik für Innere Medizin, Klinische Abteilung für Gastroenterologie und Hepatologie, Medizinische Universität Graz, Graz, Österreich; 24 Ambulante Rehabilitation, Ludwig Boltzmann Institute for Rehabilitation Research, Therme Wien Med, Wien, Österreich

**Correction:**

**Wien Klin Wochenschr 2024**

10.1007/s00508-024-02444-z

In diesem Artikel wurde der Name von dem Autor M. Idzko fälschlicherweise als M. Izdko angegeben.

Weiterhin waren die Adressdaten bzw. die Daten zur institutionellen Zugehörigkeit für K. Kastrati falsch angegeben. Statt Universitätsklinik für Innere Medizin II, Medizinische Universität Innsbruck, Innsbruck, Österreich hätte sie Universitätsklinik für Innere Medizin III, Klinische Abteilung für Rheumatologie, Medizinische Universität Wien, Wien, Österreich lauten sollen.

Außerdem waren die Adressdaten für R.H. Zwick unvollständig und hätten lauten sollen: Ambulante Rehabilitation, Ludwig Boltzmann Institute for Rehabilitation Research, Therme Wien Med, Wien, Österreich.

Der Originalartikel wurde korrigiert.

